# Manganese-Based Electrocatalysts for Acidic Oxygen Evolution: Development and Performance Evaluation

**DOI:** 10.3390/nano15181434

**Published:** 2025-09-18

**Authors:** Giulia Cuatto, Elenia De Meis, Hilmar Guzmán, Simelys Hernández

**Affiliations:** 1 CREST Group, Department of Applied Science and Technology (DISAT), Politecnico di Torino, C.so Duca degli Abruzzi, 24, 10129 Turin, Italy; giulia.cuatto@polito.it (G.C.); elenia.demeis@studenti.polito.it (E.D.M.); 2 Clean Water Center (CWC), Politecnico di Torino, C.so Duca degli Abruzzi, 24, 10129 Turin, Italy

**Keywords:** oxygen evolution reaction, Mn-based OER catalysts, catalyst optimization, catalyst stability, noble metal-free catalysts

## Abstract

Currently, the growing demand for sustainable hydrogen makes the oxygen evolution reaction (OER) increasingly important. To boost the performance of electrochemical cells for water electrolysis, both cathodic and anodic sides need to be optimized. Noble metal catalysts for the OER suffer from high costs and limited availability; therefore, developing efficient, low-cost alternatives is crucial. This work investigates manganese-based materials as potential noble-metal-free catalysts. Mn antimonates, Mn chlorates, and Mn bromates were synthesized using ultrasound-assisted techniques to enhance phase composition and homogeneity. Physicochemical characterizations were performed using X-ray diffraction (XRD) and Scanning Electron Microscopy (SEM), together with energy-dispersive X-ray spectroscopy (EDX) and surface area analyses. All samples exhibited a low surface area and inter-particle porosity within mixed crystalline phases. Among the catalysts, Mn_7.5_O_10_Br_3_, synthesized via ultrasound homogenization (30 min at 59 kHz) and calcined at 250 °C, showed the highest OER activity. Drop-casted on Fluorine-Doped Tin Oxide (FTO)-coated Ti mesh, it achieved an overpotential of 153 mV at 10 mA cm^−2^, with Tafel slopes of 103 mV dec^−1^ and 160 mV dec^−1^ at 1, 2, and 4 mA cm^−2^ and 6, 8, 10, and 11 mA cm^−2^, respectively. It also demonstrated good short-term stability (1 h) in acidic media, with a strong signal-to-noise ratio. Its short-term stability is comparable to that of the benchmark IrO_2_, with a potential drift of 15 mV h^−1^ and a standard deviation of 3 mV for the best-performing electrode. The presence of multiple phases suggests room for further optimization. Overall, this study provides a practical route for designing noble metal-free Mn-based OER catalysts.

## 1. Introduction

Global warming and environmental pollution are both closely linked to energy production, which is heavily dependent on fossil fuels. The ever-increasing energy demand of contemporary society necessitates the adaptation of the energy sector to facilitate sustainable development [1,2,3]. Hence, the focus has shifted to economical, abundant, and environmentally friendly alternatives [4]. Primary renewable energy sources, such as solar or wind energy [5], have a significant limitation due to their intermittency [6,7] and the consequent complexity of integrating them into the existing electricity grid. Therefore, electrical energy storage in green energy carriers has proven to be a promising approach [5,6]. Hydrogen (H_2_) is a lightweight and straightforward element that can store and provide usable energy [7]. Green H_2_ is obtained from water electrolysis, a key and emission-free technology that produces only H_2_ and oxygen (O_2_) as products from renewable energy sources (solar, wind, etc.) [2]. H_2_ has a high energy density (140 MJ kg^−1^, almost double that of other solid fuels, for which it is around 50 MJ kg^−1^ [8]), and it is a non-polluting energy source [7]. It is a promising renewable energy carrier [7], well-suited to the objectives and achievements of the Paris Climate Agreement, as outlined by the European Commission, which aims to achieve carbon neutrality by 2050 [9,10]. Indeed, H_2_ is suitable for both fuel and raw material applications in the chemical and petrochemical industries. Moreover, the storage efficiency of H_2_ is higher than that of batteries [2].

Water splitting is an endothermic reaction, which requires an energy input [10]. This electrolytic process involves two half-cell reactions: the H_2_ evolution reaction (HER) at the cathode and the OER at the anode. The OER is a four proton–electron-coupled process involving multi-charged transfer steps. It has a higher energy barrier and inherently slower kinetics than the HER, which makes it more difficult to occur and thus hinders H_2_ production [3].

The OER process can be carried out in a wide range of pH values, including acidic, neutral, and alkaline media [3]. Although the OER mechanism in acidic and alkaline media appears to be different, electrolysis in both basic and acidic environments involves four steps and shares the same intermediates (M-OH, M-O, and M-OOH, where M is a catalytic metal center). However, the OER in acid media is thermodynamically more limited than that in basic media due to the higher standard potential required (i.e., 1.23 and 0.4 V vs. RHE, respectively). Moreover, the OER is also kinetically sluggish in acidic environments, and most catalysts exhibit poor stability, which makes the OER challenging under acidic conditions. Nevertheless, the OER has significant advantages under acidic conditions. In addition to fewer corrosion issues, acid electrolytes enable enhanced proton conductivity, resulting in very high current densities and lower ohmic losses compared to alkaline systems [11].

Electrocatalysts help reduce the energy barrier, resulting in a greater acceleration of the reaction kinetics [10]. The essential requirements for a functional electrocatalyst are efficiency and stability [12]. Thanks to their high electrocatalytic activity towards OER in both acidic and alkaline conditions, IrO_2_ and RuO_2_ are typically used as benchmark electrocatalysts [4]. Ir and Ir-based materials are generally preferred because they are more active and stable compared to Ru, which can be unstable due to the formation of soluble oxides [13]. However, these noble metal-based electrocatalysts have significant limitations, including high cost and low material availability [14]. Those disadvantages serve as a driving force to stimulate research into new noble metal-free OER electrocatalysts [13,14,15,16]. Nevertheless, acidic media significantly affect the stability and durability of electrocatalysts, resulting in catalyst dissolution, support corrosion, and structural changes in the highly oxidative atmosphere and under the high anodic potentials required [15,16,17,18,19,20,21]. Manganese-based compounds have proven to be a promising alternative to noble metal-based electrocatalysts and other non-noble metals, such as cobalt and nickel, which are toxic (e.g., carcinogenic) [17,18,19,20,21,22,23,24].

A recent work by Delgado et al. [25] reported that Co/Mo-modified electrolytic MnO_2_ catalysts exhibit efficient OER activity in alkaline media, achieving an overpotential of 261 mV at 100 mA cm^−2^ and a low Tafel slope of 61 mV dec^−1^. Although these results cannot be directly compared with the present study, which focuses on Mn oxides in acidic conditions, both approaches highlight the versatility of Mn-based catalysts as inexpensive and efficient alternatives to noble metal-based systems. This work focuses on Mn-based compounds, aiming to develop and evaluate their OER performance in acidic conditions. As reported by the work of Pan S, Li H. et al. [26], Mn_7.5_O_10_Br_3_ and Mn_8_O_10_Cl_3_ appear to be low-cost and highly efficient OER catalysts in an acidic environment, showing an OER overpotential (η) of 295 mV at a current density of 10 mA cm^−2^, a Tafel slope (b) value of 68 mV dec^−1^, and good stability in operating conditions for at least 500 h. Therefore, Mn_7.5_O_10_Br_3_ exhibited good activity and stability, surpassing other noble metal-free catalysts [26]. MnSb_2_O_6_ has also been studied, based on the work performed by G. T. Kasun Kalhara Gunasooriya et al. [27]. There, MnSb_2_O_6_ was compared with other antimonates (such as Fe, Ni, and Co) and Mn_2_O_3_. It turned out to be the best option in terms of specific activity and stability, with the lowest change in potential during stability tests. All these mixed-oxide materials were compared with manganese oxide, showing better performance. These enhanced performances can be addressed by modifying the oxidized passivation layer, which forms on the catalyst surface under acidic conditions. Mn_7.5_O_10_Br_3_ forms a close-packed oxide surface, which seems to be related to its OER activity and stability [26], although no further studies seem to be present in terms of material characterization and optimization. Therefore, detailed exploration has been conducted, from synthesis to characterization and electrocatalytic tests. The synthesis procedure has been optimized by varying the calcination temperature, mixing procedure, and halide (Sb, Cl, or Br). Characterization of the powders was conducted using XRD, SEM, EDX, and surface area techniques to obtain powders with improved characteristics and to investigate the effect of preparation parameters on electrocatalytic performance. During the electrocatalytic tests, the activity and stability of the catalysts were evaluated [4].

**Figure 1:**
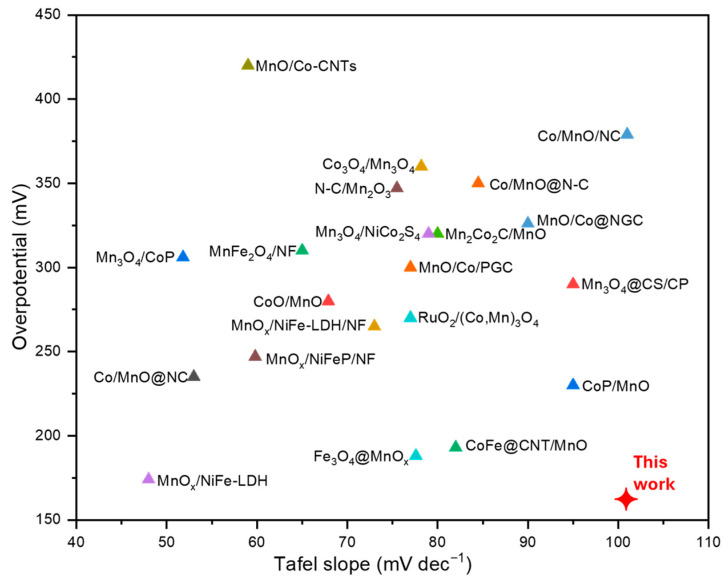
Overpotential and Tafel slope overview of the results found in the literature (numerical data extracted from [28]) and the best result obtained in this work.

Electrochemical tests demonstrated that Mn_7.5_O_10_Br_3_, obtained with a US-assisted synthesis and drop deposited on FTO-coated Ti mesh in acid media (0.5 M H_2_SO_4_), is the catalyst with the best performance, leading to an overpotential of 153 mV at 10 mA cm^−2^, showing good stability for 1 h and Tafel slope values of 103 mV dec^−1^ at 1, 2, 4 mA cm^−2^ and 160 mV dec^−1^ at 6, 8, 10, 11 mA cm^−2^. Notably, the overpotential result outperforms previously published data (see a comparison in Figure 1) with other noble metal-free catalysts (based on Mn, Co, Fe, or others), indicating the strong potential of the material, which does not contain toxic or noble metals.

## 2. Materials and Methods

### 2.1. Synthesis

Two different wet synthesis routes have been investigated to synthesize three different Mn-based powders.

To synthesize MnSb oxide, a colloidal synthesis method from the literature has been employed [27]. The process was carried out in seven steps. Firstly, the solutions were prepared, with 10 mmol of MnN_2_O_6_·4H_2_O (≥97.0%), 20 mmol of SbCl_3_ (99.0%), and 1 mL of ethylenediamine (≥99%) being dissolved in 10 mL, 10 mL, and 20 mL of ethanol, respectively. The Sb-based and Mn-based solutions were added to ethylenediamine under stirring at ambient temperature. Then, a white colloidal suspension was prepared and left to stir at ambient temperature for 24 h. The drying procedure consists of two distinct steps. Primarily, 4 pulses in the microwave, ranging from 0 W to 140 W for 30 s, were exploited as the first solvent-removal step (Multiwave Anton Paar 5000, GmbH, Graz, Austria). The humid powder obtained was further dried in static air at 200 °C for 8 h. Then, a calcination step was performed at temperatures of 400 °C, 500 °C, 600 °C, 700 °C, and 800 °C in a calcination oven (Carbolite Gero Ltd., Hope, UK). To reach the correct Mn:Sb ratio, the Sb precursor was increased in subsequent syntheses. The samples were named as MnSb_2_O_6__drying, T_calcination, and T_Sb precursor amount.

The MnCl and MnBr oxides were produced using similar syntheses [28]. A certain amount of 1.55 M MnCl_2_·4H_2_O (≥98%) or 1.66 M MnBr_2_·4H_2_O (98%) was mixed with 5 mL of 4 M MnN_2_O_6_·4H_2_O. The recipe, as reported in the literature, is presented in Table S1. Then, the solution underwent a homogenization step, either through magnetic stirring (MG) or by using an ultrasound (US) source. Different devices were used as homogenizers. A digital magnetic stirrer with a heating microprocessor (supplied by Argo Lab Srl, Carpi, Italy), an ultrasonic titanium probe (USp) (model VCX-130-220, provided by Sonics & Materials Inc., Newtown, CT, USA), and an ultrasonic bath (USb) with heating, multi-frequency, and adjustable power (model LBS2 4,5 Lt, supplied by FALC Instruments S.r.l., Treviglio, Italy). The solution was directly dried at 250 °C for 5 h, with a temperature ramp of 100 °C h^−1^. The samples were named as Mn_8_Cl_3_O_10__calcinationT or Mn_7.5_Br_3_O_10__calcinationT. The MnBr-based samples were synthesized and calcined at 250 °C, 350 °C, and 450 °C for 6 hours. Their names are Mn_7.5_Br_3_O_10__250, Mn_7.5_Br_3_O_10__350, and Mn_7.5_Br_3_O_10__450. All the synthesized samples, along with their synthesis conditions, are reported in Table S2. All the powders have been further milled to reduce the granulometry using a ball miller (purchased from Giuliani Tecnologie S.r.l., Torino, Italy) for 15 minutes at a speed of 33 m s^−1^. All reagents and solvents were purchased from Sigma-Aldrich Corporation, St. Louis, MO, USA.

### 2.2. Electrodes Preparation

The synthesized powder was converted into an ink that is compatible with the deposition methods. Nafion^TM^ 1100W (5 wt.% in lower aliphatic alcohols and water, containing 15–20% water, The Chemours Company, Wilmington, DE, USA) and 2-propanol (≥99.5%) were used as the binder and solvent, respectively, for preparing the catalytic ink. The solid phase (catalyst and the solid part of the Nafion solution) and the ratio of catalyst to solid fraction of Nafion were fixed at 3% and 30:70, respectively. The ink was sonicated with the US tip for 15 min to disperse the solid particles. A rotating glassy carbon electrode (GCE) was used to study the kinetics, to eliminate the effects of mass transfer limitations [29]. Nevertheless, after some tests, an apparent degradation of the GCE occurred. This behavior is confirmed by the study of Yi Y., Weinberg G., et al. [30]. In their work, bare, glassy carbon was subjected to OER electrochemical tests in an acidic medium (0.5 M H_2_S, pH of 0.3), and a gradual increase and shift of the anode potential were observed during linear scanning voltammetry (LSV) at high anode potentials in an acidic environment. A slight increase in current was observed at 1.2 V vs. RHE, attributed to carbon oxidation, and a second increase was noted at 1.5 V vs. RHE, resulting from the anodic oxidation of water (OER) and carbon oxidation. Furthermore, the GCE has undergone activation with respect to electrochemical reactions, leading to the formation of surface oxides on the carbon surface at high anodic potentials, as also evident from cyclic voltammetry (CV). Those results have also been confirmed by SEM-EDX analysis [30]. Therefore, herein, other supports were exploited for subsequent tests: an FTO glass substrate (7 ohm-sq) and a Titanium mesh coated with FTO (both provided by Solaronix SA, Aubonne, Switzerland). The supports were cleaned in a US bath, followed by acetone (≥99.9%), ethanol (≥99.8%), and Milli-Q. The geometrical area used for deposition was 1 cm^2^, and the remaining part of the electrode was covered with Kapton tape. At the end of the deposition, the electrode was dried at 120 °C on a hot plate for 10 minutes. Firstly, spin-coating was used to coat the FTO substrate, followed by manual drop-casting of the Ti mesh. Some electrodes were produced via spray deposition to evaluate the effect of the deposition method on the electrochemical results (see Figure S1). These droplet-based methods are cheap, fast, and easy. However, they are stochastic, making it difficult to control the repeatability and uniformity of the deposited film [31].

### 2.3. Electrochemical Procedure

A three-electrode cell at ambient temperature (T) with a Biologic VSP-300 multichannel potentiostat was used for all electrochemical tests (Bio-Logic Science Instruments SAS, Seyssinet-Pariset, Francia). A reference electrode (RE) (Ag/AgCl 3 M NaCl) and a counter electrode (CE) (Pt wire) were used. The Mn-based electrodes were connected as working electrodes (WE). The tests were conducted in an acidic environment (0.5 M H_2_SO_4_, pH of 0.3, sulfuric acid (95–97%)).

**Figure 2:**
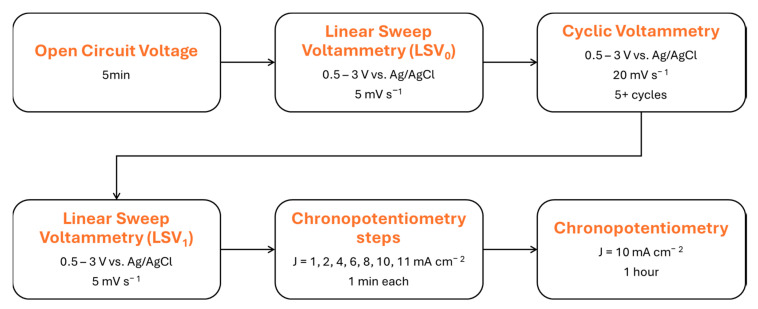
Electrochemical protocol used for OER assessment.

The electrolyte was continuously stirred with a magnetic bar at 1000 rpm throughout the entire test, after being purged for 20 minutes with N_2_. N_2_ was continuously blown into the cell at a rate of 20 mL min^−1^ throughout the entire test. The protocol is illustrated in Figure 2 and remained unchanged for all tests to ensure comparable results. An 85% compensation for ohmic losses was performed after the resistance measurement.

Overpotential (η) has been exploited as a key parameter to evaluate OER activity and compare it with a benchmark. Ideally, the potential required for a specific reaction to occur should be equal to the equilibrium potential. It is well known that the real dependency is modelled with the Nernst equation. The difference between the equilibrium potential and the real applied potential is then defined as overpotential. Furthermore, the anodic overpotential is defined as the potential difference between the applied potential and the OER equilibrium potential (E^0^ = 1.23 V vs. SHE) [32]. The overpotential is the sum of three loss sources: concentration, Ohmic, and activation (or kinetic) [33]. The overpotential value was obtained as follows: $\eta \;\left(V\right)=E_{ref}\left(V\right)-R\cdot i(A)+0.21+0.059\cdot pH-1.23\;(V)$ where $E_{ref}\left(V\right)$ is the potential vs. ref. registered during the long chronopotentiometry (CP) at 10 mA cm^−2^.

The Tafel slope was taken into account as a comparison parameter. It is a measure of electrocatalytic reaction rates. It is defined as the mV needed to increase the current by a factor of 10 and is therefore reported in mV dec^−1^ [33]. The smaller the Tafel slope, the faster the reaction rate constant, implying good electrocatalytic kinetics [13]. The Tafel slope was calculated from the chronopotentiometry at 1, 2, 4, 6, 8, 10, and 11 mA cm^−2^. Overpotentials for each step were calculated as already mentioned, and Tafel slopes were obtained by calculating the slope of the η versus Log(J) data interpolation.

For the sake of clarity and conciseness, only the key electrochemical results are presented and discussed in the main text, while the complete dataset is provided in the Supporting Information.

## 3. Results and Discussion

### 3.1. Physicochemical Characterization

#### 3.1.1. MnSb Oxides

Different synthesis batches were characterized to determine the optimal amount of Sb precursor for achieving a balanced mixture of phases in the powders (see Figure S2 for a whole view of the synthesis batches). Ultimately, the batch with the highest Sb precursor amount (2 mL of 5 M MnBr_2_·4H_2_O) was selected because it maximizes the amount of the MnSb_2_O_6_ phase. The drying procedure (microwave and drying at 200 °C for 8 h at 100 °C h^−1^) was equally maintained for all the samples. The sample prepared with the optimum Sb precursor concentration was calcined at different temperatures: 400 °C, 500 °C, 600 °C, 700 °C, and 800 °C for 5 hours at a heating rate of 100 °C h^−1^. These samples are named MnSb_2_O_6__400, MnSb_2_O_6__500, MnSb_2_O_6__600, MnSb_2_O_6__700, and MnSb_2_O_6__800, respectively.

The MnSb_2_O_6_ samples were first characterized by X-ray diffraction analysis. The spectra are reported in Figure S3. Crystalline phases such as Mn_0.667_Sb_1.333_O_4_ (ICOD 01 082 0378), Mn_2_Sb_2_O_7_ (ICOD 010 84 1236), and Mn_2_O_3_ (ICOD 01 078 0390) are recognizable in all samples. It is evident that the higher the calcination temperature, the more intense the peaks related to Mn_2_O_3_ and Mn_2_Sb_2_O_7_ are than those concerning Mn_0.667_Sb_1.333_O_4_. The XRD phase identification was carried out using the XRD pattern obtained through theoretical calculations. Referring to the XRD pattern retrieved in the literature, when MnSb_2_O_6_ is detected, the XRD pattern is not entirely reconductable for the calculated pattern (see references [34,35] and the XRD MnSb_2_O_6_ PDF pattern reported in Figure S4). That can be attributed to the high number of peaks originating from this type of powder and the preferred orientations that the particles can exhibit.

The semi-quantification of each crystalline phase (obtained from the software HighScorePlus 3.0, PANalytical B.V. 2011), the average value of the crystallite size, and the most reliable crystal system are reported in Table S3. Mn_0.667_Sb_1.333_O_4_ is always present as the main component of all the samples (45% to 70%). Mn_2_O_3_ quantity increased up to 45% for the sample calcined at 800 °C. Mn_2_Sb_2_O_7_ exhibits more intense peaks at higher temperatures, but the quantification remains relatively unchanged (~10% at 700 °C and 800 °C). This effect can be due to the increase in crystallite size at that temperature; thus, the peaks are narrower.

All the MnSb-based were characterized by EDX analyses. Oxygen, manganese, and antimony are present in all of them. Chloride was detected in trace amounts, serving as an impurity originating from the synthesis process. The calcination temperature also affects the composition. Actually, in the MnSb_2_O_6__700 and MnSb_2_O_6__800 samples, chloride was not detected. As expected, this batch contains a higher amount of antimony. On average, the percentage of antimony in all the samples is around 15–20%. It is worth noting that the composition of these samples is heterogeneous. The single-site quantification data are reported in Table S4. In particular, MnSb_2_O_6__500 exhibits a site with an Sb:Mn ratio of 1.54, which is very close to the theoretical value of 2 for the MnSb2O6 phase, in agreement with the other phases found. This sample is less homogeneous. In Table 1, the average quantification of each element (Mn, O, Sb, and Cl, when detected) is reported for each sample. The ratios between Sb, Mn, and O are reported, compared to the theoretical values for the MnSb_2_O_6_ (noted in parentheses).

An evaluation of the surface area has been performed using nitrogen physisorption with the Brunauer–Emmett–Teller (BET) method. The adsorption and desorption curves are reported in Figure S5. It can be observed that with the increasing calcination temperature, nitrogen adsorption decreases, which correlates with a decrease in the BET surface area. The values of the specific surface area (BET), total pore volume, and average pore size are reported in Table 2. Increasing the calcination temperature reduces the specific surface area, pore volume, and pore size, resulting from partial sintering and compaction of the oxide structure. The pore size of all these samples ranges between 2 nm and 50 nm; thus, these materials can be considered as mesoporous [36].

#### 3.1.2. MnCl Oxides

XRD analysis was performed to characterize the Mn_8_Cl_3_O_10_ powders. The spectra are shown in Figure S6. The spectra can be categorized into two distinct groups based on the calcination temperature of the samples. Lower calcination temperatures (250 °C to 450 °C) lead to a mixture of crystalline phases, including Mn_8_Cl_3_O_10_ (ICOD 01 081 2247), MnO_2_ (ICOD 03 065 2821), Mn_2_O_3_ (ICOD 01 071 0636), and MnO (ICOD 01 075 0625) (present only at 250 °C). On the other hand, the sample calcined at 550 °C consists primarily of Mn_2_O_3_, with a small proportion of Mn_8_Cl_3_O_10_. For that reason, higher temperatures were not tested to avoid the total evaporation of the Cl species. The phase quantification is depicted in Table 3. Among the samples, the one calcined at 350 °C contains the highest concentration of Mn_8_Cl_3_O_10_ (55%), while the sample calcined at 550 °C has the lowest amount of this phase. Furthermore, this synthesis procedure revealed that in general, a mixture of Mn oxides rather than the desired Mn_8_Cl_3_O_10_ structure is always present.

The crystallite size of the main crystalline phases was calculated using the Scherrer equation. The values are reported in Table S5. No significant variations in the crystallite size values were observed among the different calcination temperatures. All phases have crystallites with a size of less than 100 nm.

Specific surface area, total pore volume, and average pore size values of MnCl-based samples calcined from 250 °C to 450 °C are presented in Table 4. The average pore width values ranged from 2 nm to 50 nm, indicating that these samples also possess a mesoporous structure [36].

SEM analyses highlighted the heterogeneous nature of the samples (refer to Figure S7). The morphology appears to be significantly influenced by the presence of multiple phases. Thus, the most homogeneous sample is the one calcined at 550 °C, which has a high percentage of the Mn_2_O_3_ phase. The quantification obtained from XRD spectra is confirmed with EDX data. For high calcination temperatures, the chlorine content decreases to 1% (see Table 5). The heterogeneity of the samples is also confirmed. The standard deviation is high due to the presence of different phases together. Consistent with the phase quantification, the Mn_8_Cl_3_O_10__350 sample exhibits the lowest error bars, as it is primarily composed of Mn_8_Cl_3_O_10_.

#### 3.1.3. MnBr Oxides

XRD analysis was conducted to characterize, from a crystallographic perspective, the Mn_7.5_O_10_Br_3_ powders. In each sample, three different phases were found: Mn_7.5_O_10_Br_3_ (Ref. Code 01-088-1974), Mn_2_O_3_ (Ref. Code 01-078-0390), and MnO_2_ (Ref. Code 01-081-2261). All the diffractograms are reported in Figure S8. To increase the Mn_7.5_O_10_Br_3_ amount compared to the others, variations in homogenization methods, calcination temperature, and the amount of Br precursor were investigated. The sonocrystallization, i.e., the study of the effects of frequency, power, time, and shape of the US source on the crystallization of different materials, is more commonly used in the literature for the optimization of organic materials (such as cellulose, paracetamol, and fat) [37,38,39,40,41,42,43]. Therefore, the developed strategy has the potential to yield valuable results for optimizing OER catalysts.

**Figure 3:**
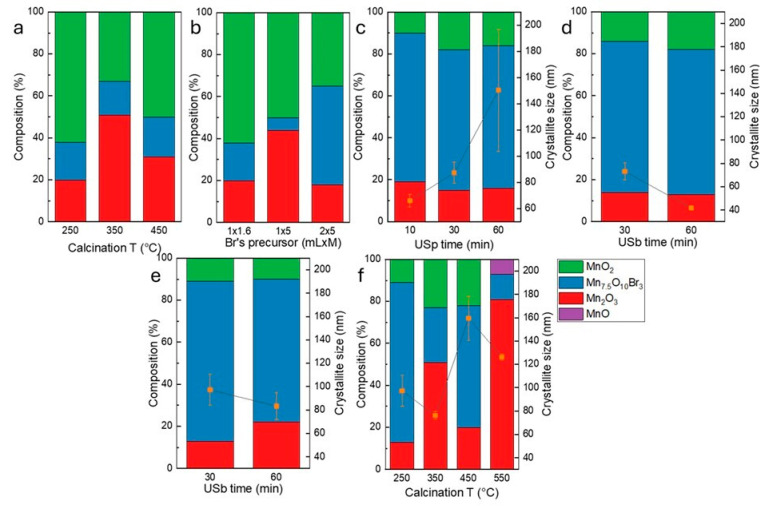
The semi-quantitative analysis made with XPert HighScore PLUS on MnBr-based powders and crystallite size obtained by Scherrer equation of (**a**) samples with 1 mL of 1.66 M Br’s precursor solution, homogenized with MG, and calcined at different T, (**b**) samples with various amount of Br’s precursor, homogenized with MG, and calcined at 250 °C, (**c**) samples with 2 mL of 5 M Br’s precursor, homogenized with US probe at 20 kHz for 10, 30, and 60 min and calcined at 250 °C, (**d**) samples with 2 mL of 5 M Br’s precursor, homogenized with US bath at 40 kHz for 30, and 60 min and calcined at 250 °C, (**e**) samples with 2 mL of 5 M Br’s precursor, homogenized with US bath at 59 kHz for 30 and 60 min and calcined at 250 °C, (**f**) samples with 2 mL of 5 M Br’s precursor, homogenized with US bath at 40 kHz for 30 min and calcined at various T.

The stacked columns graph in Figure 3 represents the crystalline phases retrieved in each sample. The samples are divided into six groups based on variation in precursor concentration, time, and frequency of homogenization source (MG, USp, and USb), as well as calcination temperature. The best results, in terms of Mn_7.5_O_10_Br_3_ percentage, were achieved with higher amounts of MnBr_2_ and by exploiting US as a homogenization source. The best result (approximately 75% of Mn_7.5_O_10_Br_3_) was obtained from the sample with the highest amount of MnBr_2_ homogenized in the US bath for 30 min at a frequency of 59 kHz. By varying the calcination temperature of this sample from 250 °C to 550 °C, no further improvements were achieved.

In terms of crystallographic planes, the most intense peaks of Mn_7.5_O_10_Br_3_ correspond to the (321), (220), and (213). In contrast, the most intense peaks of Mn_2_O_3_ and MnO_2_ correspond to the (110) and (222) planes, respectively. The MnO_2_ reflections can be attributed to β MnO_2_, as confirmed by comparison with the JCPDS card 24-0735 and supported by the recent literature [44]. All the spectra are reported in Figure S8. All the crystallite size values are reported in Table S6. It can be observed that the crystallite size changes when the frequency and timing of homogenization in the US bath are altered. In general, US-assisted homogenization helps control the crystallite size both in terms of frequency and time. At a lower frequency (20 kHz), the longer the homogenization step, the bigger the particles. Meanwhile, at higher frequencies, such as 40 and 59 kHz, the longer the duration, the smaller the particles.

**Figure 4:**
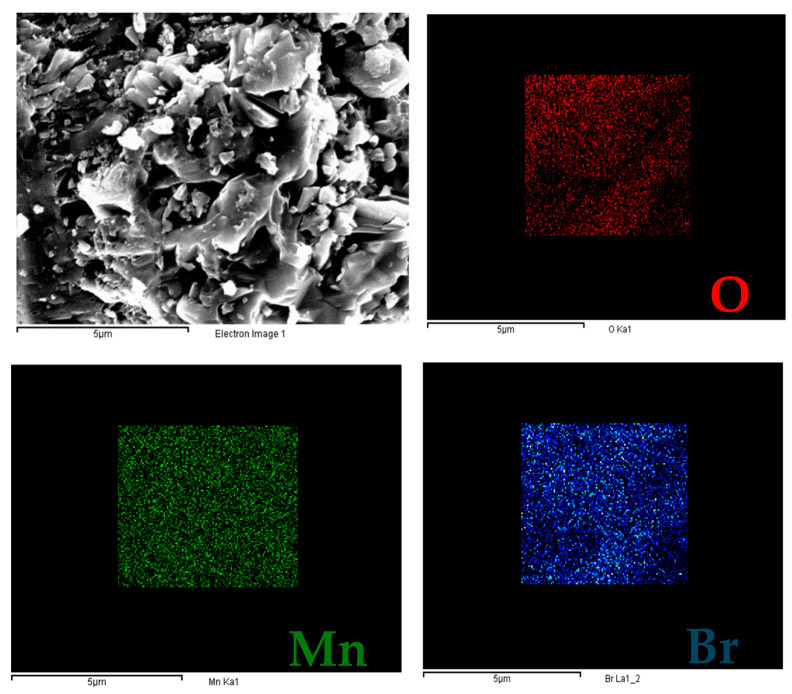
Secondary electron image of the site and maps of Mn, Br, and O of the sample Mn_7.5_O_10_Br_3__USb30min_59kHz_350.

From the SEM and EDX analyses (see Figures S9 and S10, and Table S7), the homogeneity of these samples was investigated. From a morphological perspective, the samples are not homogeneous, likely due to the presence of various phases. From EDX analyses, the results are concordant. Mn, Br, and O are the only detected elements; however, the ratios do not match the theoretical ones for Mn_7.5_O_10_Br_3_. From the EDX quantification in Table S7, the sample Mn_7.5_O_10_Br_3__USb30min_59kHz_350 exhibits a site with the ideal ratios of Mn, Br, and O. A map was created to evaluate the dispersion of the elements, as shown in Figure 4. In this site, Mn, Br, and O are very well dispersed, and the ratios are equal to 0.42, 0.32, and 0.75, almost equal to the theoretical ones (0.4, 0.3, and 0.75 for Br:Mn, Br:O, and Mn:O, respectively).

The porosity and surface area of the samples were evaluated using the BET method. From the shape of the isotherms obtained, they can be assumed to be type IV isotherms, with the resulting hysteresis phenomenon associated with capillary condensation that occurs in the mesopores [45].

**Figure 5:**
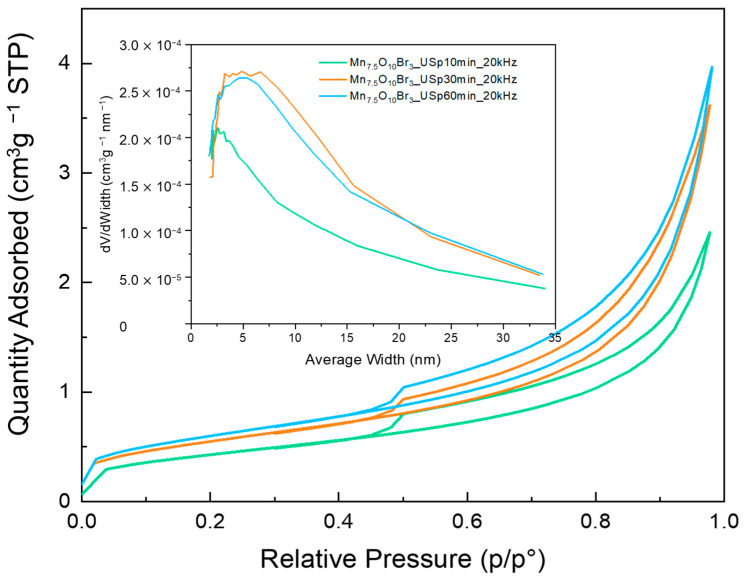
BET analysis of MnBr- oxides synthesized through US-assisted homogenization with a US probe at 20 kHz for 10, 30, and 60 min (green, orange, and blue, respectively). Main panel: nitrogen adsorption–desorption isotherms. Inset: pore size distribution curves.

The isotherms are reported in one group at a time to analyze the effect of the changing parameter on the surface area. The isotherms related to the samples obtained from homogenizing with a US probe for 10, 30, and 60 min are reported in Figure 5. All other isotherms and pore size distributions are reported in the Supporting Information Figures S11–S15. The specific surface area values are reported in Table S8. The variations in terms of specific surface area are not too significant. The area values always remain between 1 and 4 m^2^ g^−1^. From the pore size distribution graph, the peak of the pore size distribution is located near 5–10 nm, which is typical of mesopores [46]. The peak indicates a greater presence of pores around 5–10 nm, with respect to pores larger than 30 nm.

By comparing the information obtained from the XRD and BET analyses with these variables, it is evident that there is a dependency between the BET surface area and the calcination temperature (correlation graphs obtained using Principal Component Analysis in the Supporting Information, Section S3.3.1, see Figures S16–S18). Hence, the surface area increases at higher calcination temperatures, where crystallite sizes generally increase. Moreover, the surface area appears to be dependent on the amount of Br-precursor. At a higher amount of Br-precursor, then the Mn_7.5_O_10_Br_3_ content is higher and the surface area decreases. These dependencies appear to be in contrast with the sintering that occurs at higher calcination temperatures. Nevertheless, they can be attributed to the changes in phase composition of the catalyst and to the absence of visible pores as observed by SEM. Thus, the porosity detected with BET can be attributed to inter-particle porosity. On further consideration, all the evidence turns out to be consistent. Indeed, a higher Br amount leads to a lower crystallite size, primarily due to the predominance of the Mn_7.5_O_10_Br_3_ phase, resulting in better particle packaging and a lower surface area. On the other hand, a higher calcination temperature leads to larger particles, which are less capable of dense packaging together, resulting in a higher surface area.

### 3.2. Electrochemical Behavior

Electrochemical performances, including activity and stability, were first analyzed for the MnSb-oxides, then for the MnCl-oxides, and finally for the MnBr-oxides. In each group, the best samples were highlighted and then compared.

Regarding MnSb and MnCl oxides, the primary variable studied during synthesis is the calcination temperature. Regarding MnBr oxides, a comprehensive analysis was conducted, considering multiple variables, including calcination temperature, the amount of the Br precursor, homogenization conditions (source, time, and frequency), and deposition method.

**Figure 6:**
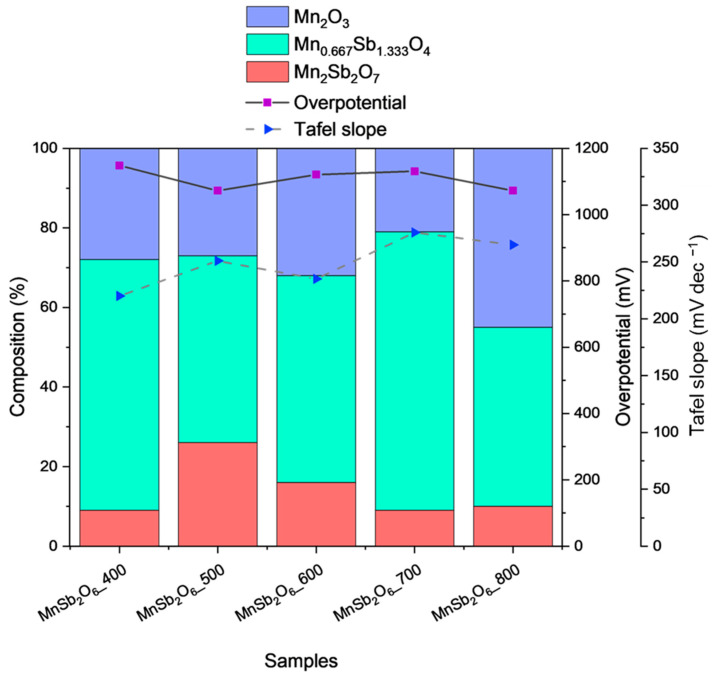
Quantification of phases of the MnSb-based catalysts calcined at different temperatures and overpotential (at 10 mA cm^−2^) and Tafel slope values. Tested in 0.5 M H_2_SO_4_.

From LSV and CV curves in Figures S19 and S20 of the MnSb samples, the higher current density at high potential values was recorded with the MnSb_2_O_6_ calcined at 600 °C (up to 40 mA cm^−2^ at 2.3 V vs. SHE). From the CVs, a similar behavior was observed for the MnSb_2_O_6_ calcined at 500 °C, 600 °C, and 800 °C. Regarding overpotential and Tafel slope values, a comparison is presented in Figure S21b and Table S9, and their relationship with the crystalline phases, as quantified via XRD analysis, is reported in Figure 6. A similar trend can be extracted from this comparison. Both overpotential and Tafel slope values depend on the content of MnSb-oxides and, especially, of Mn_2_O_3_, in agreement with the higher OER activity of α-Mn_2_O_3_ with respect to the other Mn-oxides [47,48]. These observations align with the literature reports showing that OER activity on Mn-based oxides is mediated by reversible redox cycles among Mn^2+^, Mn^3+^, and Mn^4+^. In particular, Mn^3+^ centers have been identified as especially active sites due to their favorable electronic configuration and ability to stabilize intermediates [49]. Accordingly, the enhanced performance of Mn_2_O_3_-rich samples may promote a higher density of reactive Mn^3+^ redox states, facilitating electron transfer and lowering kinetic barriers. In addition to these analyses, electrochemical impedance spectroscopy (EIS) and electrochemically active surface area (ECSA) measurements are valuable tools for deconvoluting resistance contributions and normalizing catalytic activity. Sun et al. [50] applied these methods to validate the performance of transition-metal oxides, highlighting their relevance for Mn-based systems as well. In our study, the focus was on identifying corrosion-resistant supports and establishing trends in activity and stability in acidic media; therefore, EIS and ECSA were omitted. These analyses will be addressed in future work under a flow-cell configuration, where electrode assemblies and operational conditions are more representative of practical application. It seems that the Mn_2_Sb_2_O_7_ phase is more active than the Mn_0.667_Sb_1.333_O_4_, as observed from the higher overpotential and lowest current densities of the sample calcined at 700 °C than the one at 800 °C, which contained 70% and 45% of that phase, respectively, but a similar amount of Mn_2_Sb_2_O_7_. Comparing the samples calcined at 500 °C and 800 °C, which have a similar amount of Mn_0.667_Sb_1.333_O_4_, it appears that the Mn_2_O_3_ and the Mn_2_Sb_2_O_7_ have a similar activity, since their amounts are different in these samples, but the reported overpotential at 10 mA cm^−2^ is similar. The stability was evaluated using a 30 min CP (on a GCE electrode) at 10 mA cm^−2^ (Figure S21a). It is worth noting that all samples have shown a similar stability of the reported potential throughout the entire 30-minute test duration in 0.5 M H_2_SO_4_.

**Figure 7:**
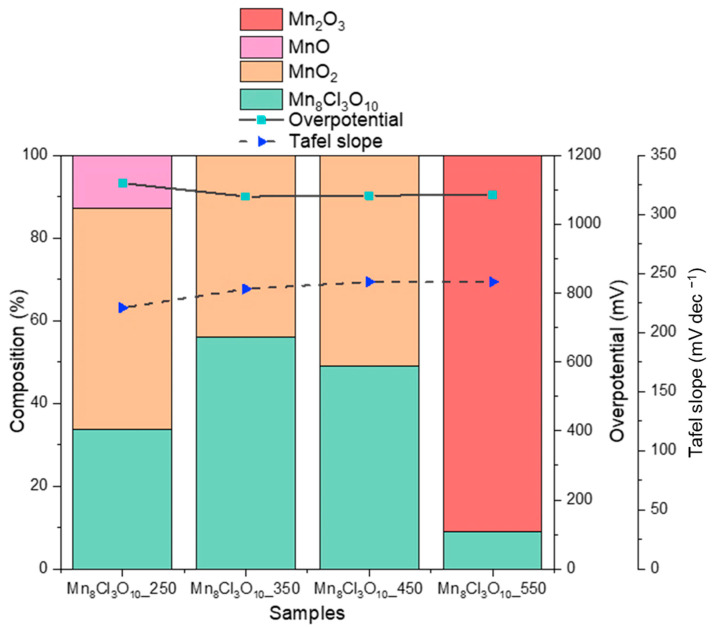
Quantification of phases of the MnCl-based catalysts calcined at different temperatures and overpotential (at 10 mA cm^−2^) and Tafel slope values. Tested in 0.5 M H_2_SO_4_.

Among the Mn-Cl samples, the Mn_8_Cl_3_O_10_ calcined at 250 °C achieved the highest current densities at high potentials in both LSV and CV tests (see Figures S22 and S23), although it had the highest onset value. The higher the calcination temperature (from 350 to 550 °C), the lower the maximum achieved current density; however, slightly lower overpotentials were obtained at all these temperatures (i.e., ~1080 mV) compared to 250 °C (i.e., 1120 mV). All the samples show a similar overpotential and stability during the CP tests at 10 mA cm^−2^ in acid media (see Figure S24a). If compared with the phase quantification obtained from XRD analysis, variations in Tafel slope and overpotential are limited to the lowest calcination temperature (see Figure 7). For the Mn_8_Cl_3_O_10__250 sample, in which the highest content of Cl (i.e., 22% at.) and the highest dimension of Mn_8_Cl_3_O_10_ crystallites (i.e., 77 nm) are present, the Tafel slope is the lowest one, i.e., 221 mV dec^−1^ (Figure S24b and Table S10). The sample at 550 °C, which reported the lowest Cl amount due to its evaporation (as expected), also reported the highest Mn_2_O_3_ amount and a similar performance (η = 1086 mV, Tafel slope = 243 mV dec^−1^) to the MnSb-sample calcined at a similar temperature of 500 °C (η = 1072 mV, Tafel slope = 251 mV dec^−1^). It indicates that the active phase is the most significant factor affecting the activity of the Mn_2_O_3_-rich samples, rather than their actual crystallite dimension (<90 nm) or surface area (<10 m^2^ g^−1^), which does not appear to have a significant impact on the OER electrochemical performance.

Regarding MnBr-oxides, a deeper investigation was conducted to optimize the synthesis and obtain a tunable catalyst. The investigated variables are calcination temperature, homogenization source (magnetic stirrer, US probe, US bath), time (10, 30, 60 min), US frequency (20, 40, 56 kHz), and deposition method (hand-sprayed or drop-deposited, with only the best-performing catalyst). The results of the electrochemical tests, compared by each single variable, are reported in the Supporting Information (from Figures S25–S42). A summary of the overpotentials at 10 mA cm^−2^ and the Tafel slopes is reported in Table 6. The Tafel slopes are reported as “slope1” and “slope2”, obtained at 1, 2, and 4 mA cm^−2^ and 6, 8, 10, and 11 mA cm^−2^, respectively. The synthesis optimization, aimed at maximizing the Mn_7.5_O_10_Br_3_, was successfully achieved with samples obtained from a higher amount of Br-precursor homogenized in a US bath for 30 and 60 min at 40 kHz and calcined at 250 °C (see Figure 3). Hence, the powders Mn_7.5_O_10_Br_3__USb30min_40kHz and Mn_7.5_O_10_Br_3__USb60min_40kHz exhibit the lowest overpotential (1902 mV) and Tafel slope1 (245 mV dec^−1^), respectively, among the tested MnBr-samples. However, those samples did not reach the performance previously reported in the literature [26], i.e., an overpotential of 295 mV at 10 mA cm^−2^ and a Tafel slope of 68 mV dec^−1^. A possible reason for this discrepancy could be attributed to the higher catalyst loading used in previous works (i.e., >7 mg cm^−1^) compared to the one that was used here in the RDE for comparison between the different synthesized materials (0.75 mg cm^−1^). Another reason could be the use of a porous and larger substrate (i.e., carbon cloth of 0.5 cm^2^), which can sustain such high catalyst loading, in contrast to the glassy carbon typically used for RDE experiments.

To assess the activity of the catalyst in a porous and conductive substrate, the Mn_7.5_O_10_Br_3__USb30min_59kHz catalyst (having the lower Tafel slope1, which means that it can sustain higher current densities) was deposited on a Ti mesh FTO covered by drop deposition and hand-spray deposition. A summary overview of the electrochemical results obtained in an acidic environment (0.5 M H_2_SO_4_, pH = 0.3) for the best sample from each group and those on Ti mesh FTO-coated is reported in the Supporting Information. The comparison in terms of LSV, CP, and Tafel slopes is reported in the Supporting Information in Figures S43–S45. The overpotential and Tafel slope values are reported in Table 7.

The deposition method and the substrate appear to be additional parameters that influence the catalyst’s performance. In the present case, the drop-casting method leads to a lower overpotential and better stability (the signal-to-noise ratio improved consistently). Regarding the stability of the drop-cast electrode, the potential drift during the test is 15 mV h^−1^, with a standard deviation of 3 mV. The potential drift was obtained as $E_{drift}\left(\mathrm{mV}\;h^{-1}\right)=(E_{final}\left(V\right)-E_{initial}\left(V\right))\cdot 1000/1\;(h)$. Some modifications to the protocols are included, as suggested by the work of Koper et al. [33]. An activation CV step was added to the protocol with a minimum number of cycles equal to 10, located around the small oxidation peak visible at 2 V vs. RHE, until its complete disappearance. The better performance of the drop-casted can be addressed by depositing a thicker layer in the electrode (with a catalyst loading around 2 mg cm^−2^), which is mechanically more stable and closer to the previous results in the literature [26]. Overall, these findings demonstrate that improvements in catalyst composition, electrode substrate, and testing conditions have a significant impact on the evaluated OER performances. Further characterization, such as X-ray Photoelectron Spectroscopy (XPS), might be useful for gaining insights into the oxidation states of Mn and the oxygen species present. The analysis of the O 1s peak, in particular, can help elucidate the origins of performance, as also highlighted by Zhang H. et al. [51]. Moreover, extending the study to different acidic pH levels would further broaden the potential application of these materials.

## 4. Conclusions

In this work, Mn-based catalysts were synthesized through different approaches, including US-assisted homogenization to enhance bromate formation over manganese oxides. Comprehensive physicochemical and electrochemical characterization has been carried out to evaluate their suitability for OER in acidic media (0.5 M H_2_SO_4_). Despite the difficulty of achieving phase-pure materials, optimization of calcination temperature and homogenization conditions led to significant improvements in phase composition and catalytic activity.

Among the evaluated systems, the most promising is the Mn_7.5_O_10_Br_3_-rich sample (almost 70% of Mn_7.5_O_10_Br_3_) obtained via ultrasound-assisted synthesis and calcination at 250 °C. Although secondary phases (MnO_2_, Mn_2_O_3_) remained, this material has achieved an overpotential of 153 mV at 10 mA cm^−2^, Tafel slopes of 103 and 160 mV dec^−1^ at low and high current densities, respectively, and a good short-term stability (1 h) when deposited by drop-casting on FTO-coated Ti mesh. These results indicate that noble metal-free manganese oxides can achieve competitive OER performance even under acidic conditions, which is typically more challenging than alkaline operation. Nevertheless, the multiphase nature of the catalysts complicates the identification of the active species and limits the establishment of structure–activity correlations. Therefore, this investigation provides a platform for further studies focused on in-depth electronic characterization (e.g., XPS) and on extended durability tests across different acidic conditions, with the ultimate goal of optimizing Mn-based oxides as efficient, low-cost, and environmentally benign OER catalysts.

## Figures and Tables

**Table 1:** EDX average concentrations in MnSb-based samples.

	MnSb_2_O_6__400	MnSb_2_O_6__500	MnSb_2_O_6__600	MnSb_2_O_6__700	MnSb_2_O_6__800
O % at.	56.88	65.77	69.90	70.24	69.96
Sb % at.	20.48	20.55	13.04	8.26	16.02
Mn % at.	20.64	13.37	16.78	21.51	14.03
Cl % at.	1.75	0.47	0.59	0	0
Sb:Mn (2)	0.99	1.54	0.78	0.38	1.14
O:Mn (6)	2.76	4.92	4.17	3.27	4.99
O:Sb (3)	2.78	3.20	5.36	8.50	4.37

**Table 2:** Specific surface area (BET), total pore volume, and average pore size of the three MnSb-based samples.

Material	BET Surface Area (m^2^ g^−1^)	Total Pore Volume (cm^3^ g^−1^)	Pore Size (nm)
MnSb_2_O_6__400	11.54 ± 0.06	0.035	12.17
MnSb_2_O_6__500	9.58 ± 0.08	0.036	15.12
MnSb_2_O_6__600	8.51 ± 0.04	0.027	12.60
MnSb_2_O_6__700	9.08 ± 0.07	0.029	12.60
MnSb_2_O_6__800	6.53 ± 0.03	0.020	12.18

**Table 3:** Quantification of phases of the MnCl-based catalysts calcined at different temperatures.

	Mn_8_Cl_3_O_10_	MnO	MnO_2_	Mn_2_O_3_
Mn_8_Cl_3_O_10__250	34%	13%	54%	-
Mn_8_Cl_3_O_10__350	56%	-	44%	-
Mn_8_Cl_3_O_10__450	49%	-	51%	-
Mn_8_Cl_3_O_10__550	9%	-	-	91%

**Table 4:** Values of specific surface area using the BET theory, total pore volume, and average pore size of the MnCl-based catalysts calcined at 250 °C, 350 °C, and 450 °C.

Catalyst	BET Surface Area (m^2^ g^−1^)	Total Pore Volume (cm^3^ g^−1^)	Pore Size (nm)
Mn_8_Cl_3_O_10__250	2.0110	0.007799	15.51351
Mn_8_Cl_3_O_10__350	2.4358	0.010372	17.03282
Mn_8_Cl_3_O_10__450	1.291	0.005241	16.23749

**Table 5:** MnCl-based samples; EDX atomic concentrations of the elements from the sampling of two sites per material.

Catalyst	O % at.	Cl % at.	Mn % at.
Mn_8_Cl_3_O_10__250	53.72 ± 8.40	22.14 ± 13.64	24.15 ± 5.24
Mn_8_Cl_3_O_10__350	52.27 ± 0.98	11.70 ± 0.04	31.04 ± 0.95
Mn_8_Cl_3_O_10__450	38.76 ± 22.11	13.77 ± 6.07	47.48 ± 16.04
Mn_8_Cl_3_O_10__550	32.54 ± 25.82	1.13 ± 0.27	66.34 ± 26.09

**Table 6:** Overpotential at 10 mA cm^−2^ and Tafel slope values at low and high current densities for all Mn_7.5_O_10_Br_3_ tested samples. Tested in 0.5 M H_2_SO_4_.

Samples	Overpotential, η (mV) @ 10 mA cm^−2^	Tafel Slope, b (mV dec^−1^)	
		Slope_1_ (mA cm^−2^)	Slope_2_ (mA cm^−2^)
Mn_7.5_O_10_Br_3__250	2003	273	401
Mn_7.5_O_10_Br_3__350	2213	289	364
Mn_7.5_O_10_Br_3__450	2103	283	391
Mn_7.5_O_10_Br_3__250sat	1982	263	397
Mn_7.5_O_10_Br_3__250dsat	2122	283	365
Mn_7.5_O_10_Br_3__USp10min_20kHz	1903	263	331
Mn_7.5_O_10_Br_3__USp30min_20kHz	2221	275	392
Mn_7.5_O_10_Br_3__USp60min_20kHz	2131	266	375
Mn_7.5_O_10_Br_3__USb30min_40kHz	1930	282	331
Mn_7.5_O_10_Br_3__USb60min_40kHz	1902	260	373
Mn_7.5_O_10_Br_3__USb30min_59kHz	2155	245	378
Mn_7.5_O_10_Br_3__USb60min_59kHz	2113	294	334
Mn_7.5_O_10_Br_3__USb30min_59kHz_350	2223	307	404
Mn_7.5_O_10_Br_3__USb30min_59kHz_450	1882	266	338
Mn_7.5_O_10_Br_3__USb30min_59kHz_550	1828	286	336

**Table 7:** Values of overpotential (η, mV) and Tafel slope (mV dec^−1^) for drop-casting_acidpH, hand-spray_acidpH, and bareTimeshFTOcoated samples. Tested in 0.5 M H_2_SO_4_.

Samples	Overpotential, η (mV) @ 10 mA cm^−2^	Tafel Slope, b (mV dec^−1^)	
		Slope_1_ (mA cm^−2^)	Slope_2_ (mA cm^−2^)
Drop-casting_acid pH	153	103	160
Hand-spray_acid pH	593	215	264

## Data Availability

The data presented in this study are available on request from the corresponding author.

## Supplementary Materials

The following supporting information can be downloaded at: https://www.mdpi.com/article/10.3390/nano15181434/s1, Table S1: main values of the two precursors (MnBr_2_∙4H_2_O and Mn(NO_3_)_2_∙4H_2_O) used in the literature [26]; Table S2: list of all the samples synthesized with their respective synthesis conditions; Figure S1: a schematic explanation of Ti-mesh FTO coated with Mn-based deposited ink with the presence of Kapton tape; B. Ti-mesh FTO coated with Mn-based deposited ink; Figure S2: semi-quantitative percentage of the crystalline phases present in all the synthesized powders. The first five powders are the first attempts, where different amounts of Br-precursor have been used in order to maximize the MnBr-oxide phase; Figure S3: XRD spectra of MnSb-based oxides calcined at 400 °C, 500 °C, 600 °C, 700 °C, and 800 °C for 5 h with a temperature ramp equal to 100 °C h^−1^; Table S3: average crystallite size (nm), semi-quantification, and crystal system relatable to each crystalline phase for all the samples; Table S4: quantification through EDX analysis of MnSb-based samples calcined at 400 °C, 500 °C, 600 °C, 700 °C, and 800 °C, 2 or 4 sites each sample, oxygen, antimony, manganese, and chlorine atomic percentage. Ratios between Sb and Mn, O and Mn, and O and Sb. For each sample, the average and error on quantification and ratios are reported; Figure S4: XRD spectrum of the pure MnSb_2_O_6_ crystalline phase. This spectrum is the reference pattern reported in the HighScore Plus library (ICSD74380); Figure S5: BET curves of adsorption and desorption of all the MnSb-based samples from the Sb-richest batch; Figure S6: XRD spectra of MnCl-based catalysts synthesized and calcined at 250 °C, 350 °C, 450 °C, and 550 °C. The peaks are indexed as Mn_8_Cl_3_O_10_, MnO_2_, MnO, or Mn_2_O_3_; Table S5: crystallite size from XRD data by using the Scherrer equation of the MnCl-based catalysts calcined at different temperatures; Figure S7: SEM images (magnification 10k) of various sites of the MnCl-based catalysts. a. Mn_8_Cl_3_O_10_ calcined at 250 °C; b. Mn_8_Cl_3_O_10_ calcined at 350 °C; c. Mn_8_Cl_3_O_10_ calcined at 450 °C; and d. site 1 of Mn_8_Cl_3_O_10_ calcined at 550 °C with 20k magnification; Figure S8: XRD patterns of some Mn_7.5_O_10_Br_3_ samples; Table S6: average crystallite size of Mn_7.5_O_10_Br_3_; Figure S9: SEM images A1 Mn_7.5_O_10_Br_3_ _USp10min_20kHz, A2. Mn_7.5_O_10_Br_3_ _USp30min_20kHz, A3. Mn_7.5_O_10_Br_3_ _USp60min_20kHz, A4. Mn_7.5_O_10_Br_3_ _USb30min_40kHz, A5. Mn_7.5_O_10_Br_3_ _USb60min_40kHz, A6. Mn_7.5_O_10_Br_3_ _USb30min_59kHz, A7. Mn_7.5_O_10_Br_3_ _USb60min_59kHz with magnification; Table S7: percentage atomic values of elements (Mn, Br, and O) in the samples; Figure S10: SEM-EDX images of one site of Mn_7.5_O_10_Br_3_ _USb30 min_59kHz_350 sample; Figure S11: BET analysis of nitrogen adsorption–desorption isotherms for Mn_7.5_O_10_Br_3_ catalysts by varying the calcination T; Figure S12: BET analysis of nitrogen adsorption–desorption isotherms for Mn_7.5_O_10_Br_3_ catalysts by varying the precursors’ concentration; Figure S13: B3. BET analysis of nitrogen adsorption–desorption isotherms and B4. pore size distribution curves (inserted images) by varying USp and USb frequencies at constant sonication t of 30 min; Figure S14: B5. BET analysis of nitrogen adsorption–desorption isotherms and B6. pore size distribution curves (inserted images) by varying USp and USb frequencies at constant sonication t of 60 min; Figure S15: B7. BET analysis of nitrogen adsorption–desorption isotherms and B8. pore size distribution curves (inserted images) by varying calcination T; Table S8: BET surface area of all samples; Figure S16: cumulative variance depending on the number of principal components considered; Figure S17: effect of Br-precursor amount (in moles, 4.8, 5, and 10 moles). The axes represent the samples (in order as presented in this work) vs. principal component 2; Figure S18: Q residuals plot; Figure S19: RDE test, LSV1 curves of MnSb-based catalysts calcined at different temperatures. Catalyst loading 0.75 mg cm^−2^, reference electrode Ag/AgCl, counter electrode Pt wire. Potential range 0–2.3 V vs. SHE. Tested in 0.5 M H_2_SO_4_; Figure S20: RDE test, CV curves of MnSb-based catalysts calcined at different temperatures. Catalyst loading 0.75 mg cm^−2^, reference electrode Ag/AgCl, counter electrode Pt wire. Potential range 0–2.15 V vs. SHE. a. whole curves, b. magnification from 1.4 to 2.6 V. Tested in 0.5 M H_2_SO_4_; Figure S21: RDE test, a. CP curves of MnSb-based catalysts calcined at different temperatures and 10 mA cm^−2^, b. Tafel plots of MnSb-based catalysts calcined at different temperatures. Catalyst loading: 0.75 mg cm^−2^, reference electrode Ag/AgCl, counter electrode Pt wire. Tested in 0.5 M H_2_SO_4_; Table S9: RDE test, overpotential at 10 mA cm^−2^ and Tafel slope values of MnSb-based catalysts calcined at different temperatures. Tested in 0.5 M H_2_SO_4_; Figure S22: RDE test, LSV1 curves of MnCl-based catalysts calcined at different temperatures. Catalyst loading: 0.75 mg cm^−2^, reference electrode Ag/AgCl, counter electrode Pt wire. Potential range 0–2.3 V vs. SHE. Tested in 0.5 M H_2_SO_4_; Figure S23: RDE test, CV curves of MnCl-based catalysts calcined at different temperatures. Catalyst loading: 0.75 mg cm^−2^, reference electrode Ag/AgCl, counter electrode Pt wire. Potential range 0–2.15 V vs. SHE. a. whole curves, b. magnification from 1.4 to 2.6 V vs. SHE and from 0 to 25 mA cm^−2^. Tested in 0.5 M H_2_SO_4_; Figure S24: RDE test, a. CP curves of MnCl-based catalysts calcined at different temperatures and 10 mA cm^−2^, b. Tafel plots of MnSb-based catalysts calcined at different temperatures. Catalyst loading: 0.75 mg cm^−2^, reference electrode Ag/AgCl, counter electrode Pt wire. Potential range 0–2.15 V vs. SHE. Tested in 0.5 M H_2_SO_4_; Table S10: RDE test, overpotential at 10 mA cm^−2^ and Tafel slope values of MnSb-based catalysts calcined at different temperatures. Tested in 0.5 M H_2_SO_4_; Figure S25: LSV curves for Mn_7.5_O_10_Br_3_ calcined at 250 °C, 350 °C and 450 °C at 5 mV s^−1^ in 0.5 M H_2_SO_4_. Tested in 0.5 M H_2_SO_4_; Figure S26: CP curves for Mn_7.5_O_10_Br_3_ calcined at 250 °C, 350 °C and 450 °C at 10 mA cm^−2^ in 0.5 M H_2_SO_4_. Tested in 0.5 M H_2_SO_4_; Figure S27: Tafel plots for Mn_7.5_O_10_Br_3_ calcined at 250 °C, 350 °C and 450 °C in 0.5 M H_2_SO_4_; Figure S28: LSV curves for Mn_7.5_O_10_Br_3_ _250, Mn_7.5_O_10_Br_3__250sat and Mn_7.5_O_10_Br_3_ _250dsat at 5 mV s^−1^. Tested in 0.5 M H_2_SO_4_; Figure S29: CP curves for Mn_7.5_O_10_Br_3_ _250, Mn_7.5_O_10_Br_3_ _250sat and Mn_7.5_O_10_Br_3_ _250dsat samples at 10 mA cm^−2^. Tested in 0.5 M H_2_SO_4_; Figure S30: Tafel plots for Mn_7.5_O_10_Br_3_ _250, Mn_7.5_O_10_Br_3_ _250sat and Mn_7.5_O_10_Br_3_ _250dsat. Tested in 0.5 M H_2_SO_4_; Figure S31: LSV curves for Mn_7.5_O_10_Br_3_ _USp10min_20kHz, Mn_7.5_O_10_Br_3_ _USp30min_20kHz and Mn_7.5_O_10_Br_3_ _USp60min_20kHz samples at 5 mV s^−1^. Tested in 0.5 M H_2_SO_4_; Figure S32: CP curves for Mn_7.5_O_10_Br_3_ _USp10min_20kHz, Mn_7.5_O_10_Br_3_ _USp30min_20kHz and Mn_7.5_O_10_Br_3_ _USp60min_20kHz samples at 10 mA cm^−2^. Tested in 0.5 M H_2_SO_4_; Figure S33: Tafel plots for Mn_7.5_O_10_Br_3__USp10min_20kHz, Mn_7.5_O_10_Br_3__USp30min_20kHz and Mn_7.5_O_10_Br_3__USp60min_20kHz. Tested in 0.5 M H_2_SO_4_, Figure S34: LSV curves for Mn_7.5_O_10_Br_3__USp30min_20kHz, Mn_7.5_O_10_Br_3__USp30min_40kHz, and Mn_7.5_O_10_Br_3__USp30min_59kHz at 5 mV s^−1^. Tested in 0.5 M H_2_SO_4_; Figure S35: CP curves for Mn_7.5_O_10_Br_3__USp30min_20kHz, Mn_7.5_O_10_Br_3__USb30min_40kHz, and Mn_7.5_O_10_Br_3__USb30min_59kHz samples at 10 mA cm^−2^. Tested in 0.5 M H_2_SO_4_; Figure S36: Tafel plots for Mn_7.5_O_10_Br_3__USp30min_20kHz, Mn_7.5_O_10_Br_3__USp30min_40kHz, and Mn_7.5_O_10_Br_3__USp30min_59kHz. Tested in 0.5 M H_2_SO_4_; Figure S37: CP curves for Mn_7.5_O_10_Br_3__USp60min_20kHz, Mn_7.5_O_10_Br_3__USb60min_40kHz, and Mn_7.5_O_10_Br_3__USb60min_59kHz samples at 10 mA cm^−2^. Tested in 0.5 M H_2_SO_4_; Figure S38: LSV curves for Mn_7.5_O_10_Br_3__USp60min_20kHz, Mn_7.5_O_10_Br_3__USp60min_40kHz, and Mn_7.5_O_10_Br_3__USp60min_59kHz at 5 mV s^−1^. Tested in 0.5 M H_2_SO_4_; Figure S39: Tafel plots for Mn_7.5_O_10_Br_3__USp60min_20kHz, Mn_7.5_O_10_Br_3__USb60min_40kHz, and Mn_7.5_O_10_Br_3__USb60min_59kHz samples. Tested in 0.5 M H_2_SO_4_; Figure S40: LSV curves for Mn_7.5_O_10_Br_3__USb30min_59kHz_250, Mn_7.5_O_10_Br_3__USb30min_59kHz_350, Mn_7.5_O_10_Br_3__USb30min_59kHz_450, and Mn_7.5_O_10_Br_3__USb30min_59kHz_550 samples at 5 mV s^−1^. Tested in 0.5 M H_2_SO_4_; Figure S41: CP curves for Mn_7.5_O_10_Br_3__USb30min_59kHz, Mn_7.5_O_10_Br_3__USb30min_59kHz_350, Mn_7.5_O_10_Br_3__USb30min_59kHz_450, and Mn_7.5_O_10_Br_3__USb30min_59kHz_550 samples at 10 mA cm^−2^. Tested in 0.5 M H_2_SO_4_; Figure S42: Tafel plots for Mn_7.5_O_10_Br_3__USb30min_59kHz, Mn_7.5_O_10_Br_3__USb30min_59kHz_350, Mn_7.5_O_10_Br_3__USb30min_59kHz_450, and Mn_7.5_O_10_Br_3__USb30min_59kHz_550 samples. Tested in 0.5 M H_2_SO_4_; Figure S43: CP curves for drop-casting_acidpH, hand-spray_acidpH, and bareTimeshFTOcoated samples at 10 mA cm^−2^. Tested in 0.5 M H_2_SO_4_; Figure S44: Tafel plots for drop-casting_acidpH, hand-spray_acidpH, and bareTimeshFTOcoated samples. Tested in 0.5 M H_2_SO_4_; Figure S45: CP curves for Mn_7.5_O_10_Br_3__250, Mn_7.5_O_10_Br_3__250dsat, Mn_7.5_O_10_Br_3__USp10min_20kHz, Mn_7.5_O_10_Br_3__USp60min_20kHz, Mn_7.5_O_10_Br_3__USb30min_59kHz, Mn_7.5_O_10_Br_3__USb30min_59kHz_550, hand-spray_acidpH, and drop-casting_acidpH samples at 10 mA cm^-2^. Tested in 0.5 M H_2_SO_4_; Figure S46: Tafel plots for Mn_7.5_O_10_Br_3__250, Mn_7.5_O_10_Br_3__250dsat, Mn_7.5_O_10_Br_3__USp10min_20kHz, Mn_7.5_O_10_Br_3__USp60min_20kHz, Mn_7.5_O_10_Br_3__USb30min_59kHz, Mn_7.5_O_10_Br_3__USb30min_59kHz_550, hand-spray_acidpH, and drop-casting_acidpH samples. Tested in 0.5 M H_2_SO_4_; Table S11: overpotential and Tafel slope values for the best sample of each group.
