# Supplementary material for: Manganese-Based Electrocatalysts for Acidic Oxygen Evolution: Development and Performance Evaluation
Source: Nanomaterials (Basel). 2025 Sep 18;15(18):1434. doi: 10.3390/nano15181434 (PMC12472920; doi:10.3390/nano15181434)
Supplement: Supplementary file 1 [file nanomaterials-15-01434-s001.zip › nanomaterials-3835935-supplementary.pdf]

Manganese-Based Electrocatalysts for Acidic Oxygen Evolution: Development and Performance Evaluation

Giulia Cuatto [1], Elenia De Meis [1], Hilmar Guzmán [1,*] and Simelys Hernández [1,2,*]

[1] CREST Group, Department of Applied Science and Technology (DISAT), Politecnico di Torino, C.so Duca degli Abruzzi, 24, 10129 Turin, Italy; giulia.cuatto@polito.it (G.C.); elenia.demeis@studenti.polito.it (E.D.M.)

[2] Clean Water Center (CWC), Politecnico di Torino, C.so Duca degli Abruzzi, 24, 10129 Turin, Italy

* Correspondence: hilmar.guzman@polito.it (H.G.); simelys.hernandez@polito.it (S.H.)

Supporting Information

Summary

Manganese-based Electrocatalysts for Acidic Oxygen Evolution: Development and Performance Evaluation. 1

1. Synthesis 2
2. Electrode preparation 3
3. Results and discussion 4
   - 3.1. MnSb oxides 4
   - 3.2. MnCl- oxides 9
   - 3.3. MnBr-oxides 11
     - 3.3.1. Principal Component Analysis (PCA) 18
   - 3.4. Electrochemical characterization 20

1. Synthesis

Firstly, the solutions with the two precursors must be prepared. The quantities of precursors, the volume, and the concentration of the solutions are summarized in **Table S1**.

Table S1. *Main values of the two precursors ($MnBr_2{\cdot}4H_2O$ and $Mn(NO_3)_2{\cdot}4H_2O$) used in literature*[13].

	$MnBr_2{\cdot}4H_2O$	$Mn(NO_3)_2{\cdot}4H_2O$
**m(g)**	1.43	5.02
**V (mL)**	3.00	5.00
**C (M)**	1.67	4.00

The first samples were obtained by using a magnetic stirrer for 10 min at 500 rpm. Later, a US probe at 20 kHz (130 W), for a time varying between t=10, 30 or 60 min and a US bath at 40 or 59 kHz (260 W) for a time between t=30 or 60 min were used to improve the homogenization process.

The resulting samples from these syntheses have been grouped based on precursors' concentration, homogenization source (magnetic stirring (MG), US probe (USp), and US bath (USb)), duration and calcination temperature. All the samples with their synthesis conditions are reported in **Table S2**.

Table S2. *List of all the samples synthesized with their respective synthesis conditions.*

Sample name	$MnBr_2{\cdot}4H_2O$		Homogenization			Calcination Temperature (°C)
	C (M)	V (mL)	Source	Time (min)	Conditions	
$Mn_{7.5}O_{10}Br_3$_250	1.6	3	MG	10	500 rpm	250
$Mn_{7.5}O_{10}Br_3$_350	1.6	3	MG	10	500 rpm	350
$Mn_{7.5}O_{10}Br_3$_450	1.6	3	MG	10	500 rpm	450
$Mn_{7.5}O_{10}Br_3$_250sat	5	1	MG	10	500 rpm	250
$Mn_{7.5}O_{10}Br_3$_250dsat	5	2	MG	10	500 rpm	250
$Mn_{7.5}O_{10}Br_3$_USp10min_20kHz	5	2	USp	10	20 kHz, 130 W	250
$Mn_{7.5}O_{10}Br_3$_USp30min_20kHz	5	2	USp	30	20 kHz, 130 W	250
$Mn_{7.5}O_{10}Br_3$_USp60min_20kHz	5	2	USp	60	20 kHz, 130 W	250
$Mn_{7.5}O_{10}Br_3$_USb30min_40kHz	5	2	USb	30	40 kHz, 260 W	250
$Mn_{7.5}O_{10}Br_3$_USb60min_40kHz	5	2	USb	60	40 kHz, 260 W	250
$Mn_{7.5}O_{10}Br_3$_USb30min_59kHz_250	5	2	USb	30	59 kHz, 260 W	250
$Mn_{7.5}O_{10}Br_3$_USb60min_59kHz_250	5	2	USb	60	59 kHz, 260 W	250
$Mn_{7.5}O_{10}Br_3$_USb30min_59kHz_350	5	2	USb	30	59 kHz, 260 W	350
$Mn_{7.5}O_{10}Br_3$_USb30min_59kHz_450	5	2	USb	30	59 kHz, 260 W	450

2. Electrode preparation

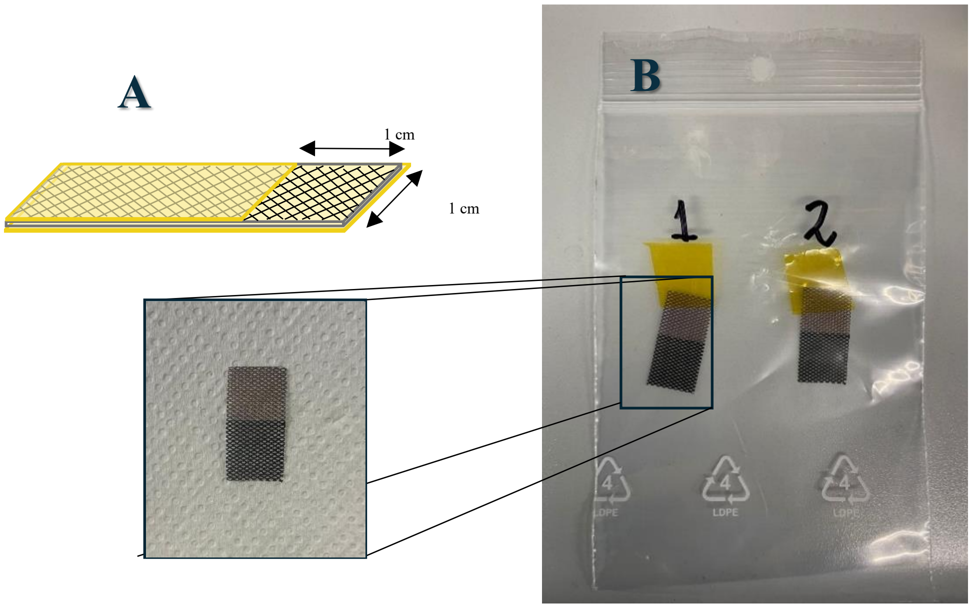

Figure S1. *A. schematic explanation Ti-mesh FTO coated with Mn-based deposited ink with the presence of Kapton tape. B. Ti-mesh FTO coated with Mn-based deposited ink*

3. Results and discussion

3.1. MnSb oxides

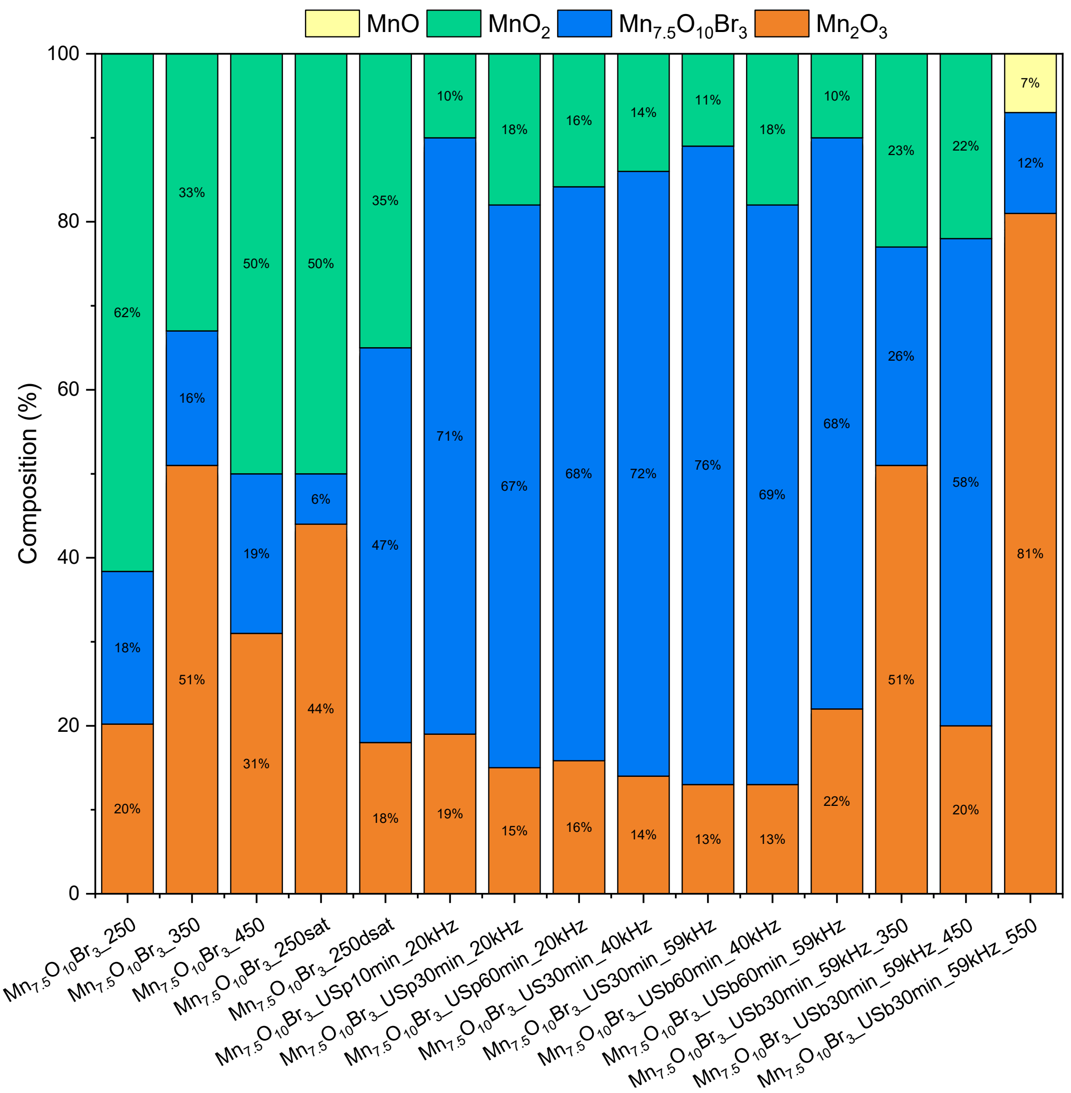

Figure S2: Semiquantitative percentage of the crystalline phases present in all the synthesized powders. The first 5 powders are the first attempts, where different amount of Br-precursor has been used in order to maximize the MnBr-oxide phase.

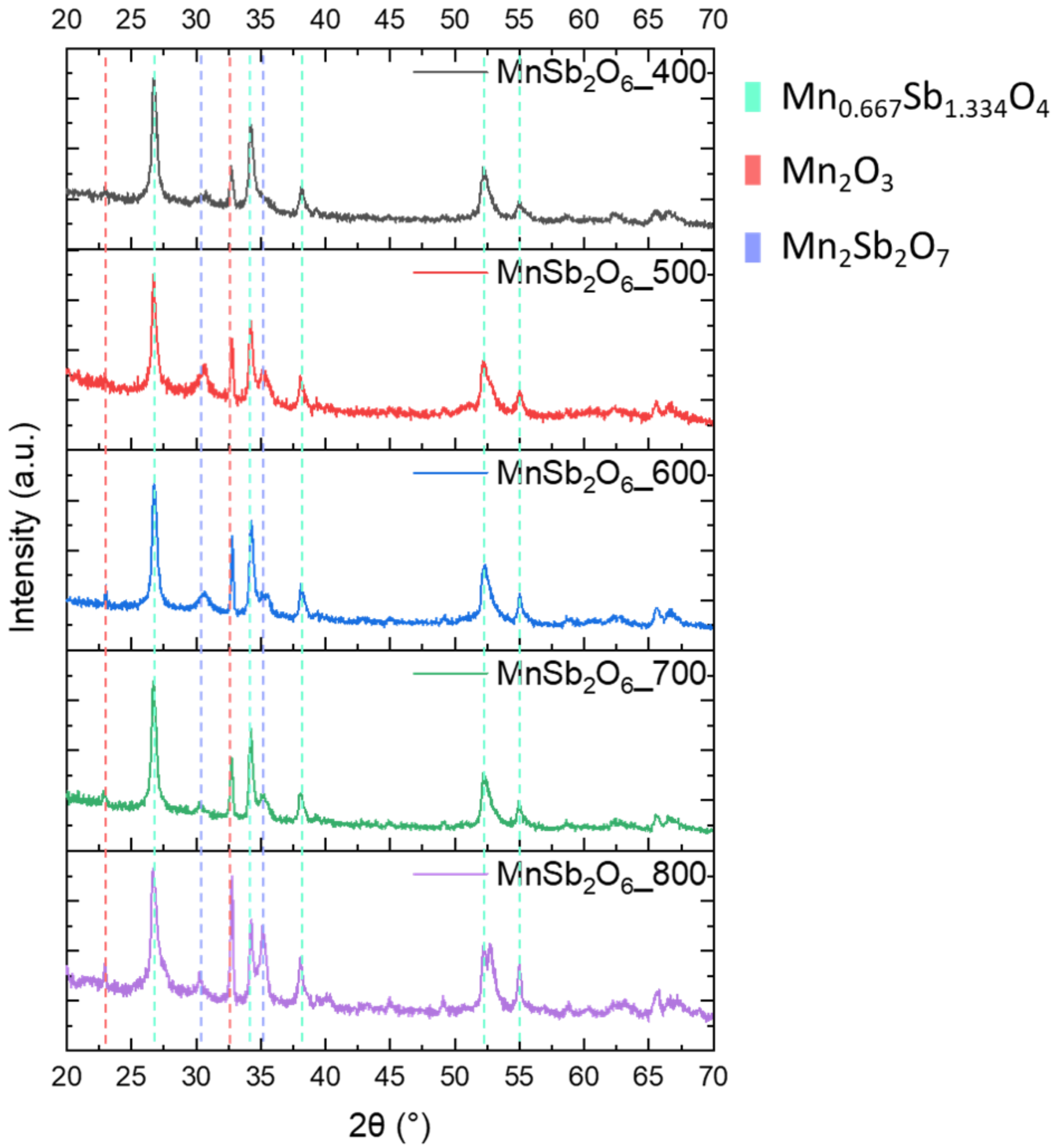

Figure S3: XRD spectra of MnSb-based oxydes calcined at 400°C, 500 °C, 600 °C, 700 °C, and 800 °C for 5 h with a temperature ramp equal to 100 °C h-1.

Table S3: Average crystallite size (nm), semi-quantification and crystal system relatable to each crystalline phase for all the samples.

MnSb$_2$O$_6$_400			
**Crystalline phase**	**Quantification**	**Crystallite size (nm)**	**Crystal system**
**Mn$_{0.667}$Sb$_{1.333}$O$_4$**	63 %	21.9	Tetragonal
**Mn$_2$Sb$_2$O$_7$**	9 %	6.1	Hexagonal
**Mn$_2$O$_3$**	28 %	25.5	Cubic
**MnSb$_2$O$_6$_500**			
**Crystalline phase**	**Quantification**	**Crystallite size (nm)**	**Crystal system**
**Mn$_{0.667}$Sb$_{1.333}$O$_4$**	47 %	20.7	Tetragonal
**Mn$_2$Sb$_2$O$_7$**	26 %	7.9	Hexagonal
**Mn$_2$O$_3$**	27 %	23.0	Cubic
**MnSb$_2$O$_6$_600**			
**Crystalline phase**	**Quantification**	**Crystallite size (nm)**	**Crystal system**
**Mn$_{0.667}$Sb$_{1.333}$O$_4$**	52 %	26.9	Tetragonal
**Mn$_2$Sb$_2$O$_7$**	16 %	5.4	Hexagonal
**Mn$_2$O$_3$**	32 %	31.2	Cubic
**MnSb$_2$O$_6$_700**			
**Crystalline phase**	**Quantification**	**Crystallite size (nm)**	**Crystal system**
**Mn$_{0.667}$Sb$_{1.333}$O$_4$**	70 %	22.4	Tetragonal
**Mn$_2$Sb$_2$O$_7$**	9 %	8.3	Hexagonal
**Mn$_2$O$_3$**	22 %	26.4	Cubic
**MnSb$_2$O$_6$_800**			
**Crystalline phase**	**Quantification**	**Crystallite size (nm)**	**Crystal system**
**Mn$_{0.667}$Sb$_{1.333}$O$_4$**	45 %	21.3	Tetragonal
**Mn$_2$Sb$_2$O$_7$**	10 %	12.9	Hexagonal
**Mn$_2$O$_3$**	45 %	45.9	Cubic

Table S4: Quantification through EDX analysis of MnSb-based samples calcined at 400 °C, 500 °C, 600 °C, 700 °C, and 800 °C, 2 or 4 sites each sample, oxygen, antimony, manganese, and chlorine atomic percentage. Ratios between Sb and Mn, O and Mn, and O and Sb. For each sample the average and error on quantification and ratios are reported.

	$MnSb_2O_6$_400						***$MnSb_2O_6$_500***				***$MnSb_2O_6$_600***			
**Sampling**	**s1**	**s2**	**s3**	**s4**	**Mean**	**St. Dev.**	**s1**	**s2**	**Mean**	**St. Dev.**	**s1**	**s2**	**Mean**	**St. Dev.**
**O %at**	60.46	40.22	59.35	67.50	56.88	11.68	70.69	60.85	65.77	6.96	69.58	70.21	69.90	0.45
**Sb %at**	18.22	29.43	17.90	16.36	20.48	6.02	17.62	23.48	20.55	4.14	14.27	11.80	13.04	1.75
**Mn %at**	19.71	27.36	21.57	13.93	20.64	5.54	11.56	15.17	13.37	2.55	16.15	17.40	16.78	0.88
**Cl %at**	1.60	2.99	1.18	1.23	1.75	0.85	0.43	0.50	0.47	0.05	0.00	0.59	0.30	0.42
**Sb:Mn ratio =2**	0.92	1.08	0.83	1.17	0.99	0.15	1.52	1.55	1.54	0.02	0.88	0.68	0.78	0.15
**O:Mn ratio =6**	3.07	1.47	2.75	4.85	2.76	1.39	6.12	4.01	4.92	1.49	4.31	4.04	4.17	0.19
**O:Sb ratio =3**	3.32	1.37	3.32	4.13	2.78	1.17	4.01	2.59	3.20	1.00	4.88	5.95	5.36	0.76
	$MnSb_2O_6$_700				***$MnSb_2O_6$_800***									
**Sampling**	**s1**	**s2**	**Mean**	**St. Dev.**	**s1**	**s2**	**s3**	**s4**	**Mean**	**St. Dev.**				
**O %at**	69.42	71.05	70.24	1.15	68.94	69.43	70.02	71.43	69.96	1.08				
**Sb %at**	3.34	13.18	8.26	6.96	16.54	18.92	10.98	17.63	16.02	3.50				
**Mn %at**	27.24	15.77	21.51	8.11	14.52	11.65	19.00	10.93	14.03	3.66				
**Cl %at**	0.00	0.00	0.00	-	0.00	0.00	0.00	0.00	0.00	-				
**Sb:Mn ratio =2**	0.12	0.84	0.38	0.50	1.14	1.62	0.58	1.61	1.14	0.50				
**O:Mn ratio =6**	2.55	4.51	3.27	1.38	4.75	5.96	3.69	6.54	4.99	1.27				
**O:Sb ratio =3**	20.78	5.39	8.50	10.88	4.17	3.67	6.38	4.05	4.37	1.23				

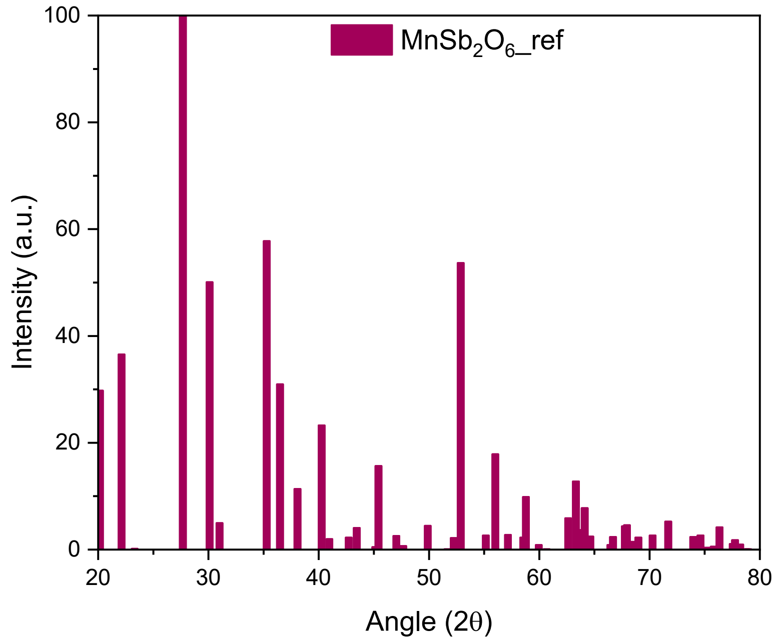

Figure S4: XRD spectrum of the pure $MnSb_2O_6$ crystalline phase. This spectrum is the reference pattern reported in the HighScore Plus library (ICSD74380).

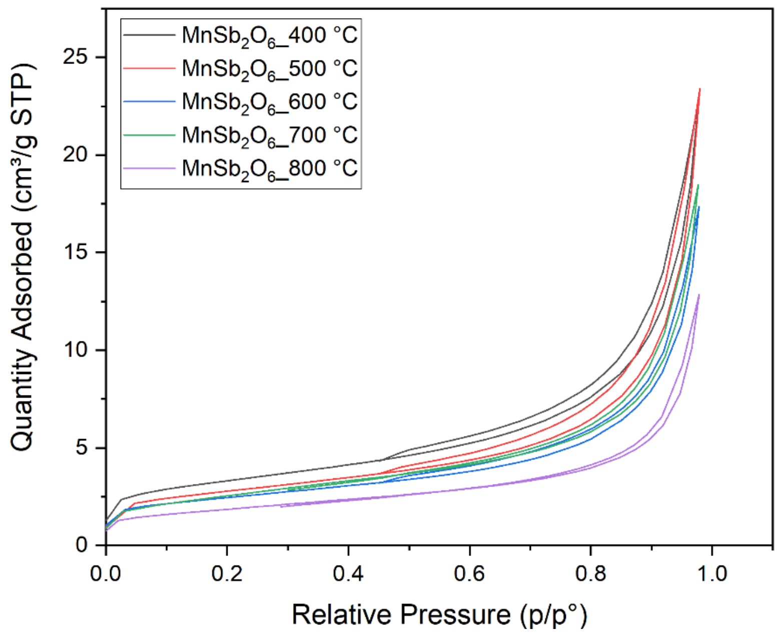

Figure S5: BET curves of adsorption and desorption of all the MnSb-based samples from the Sb-richest batch.

3.2. MnCl- oxides

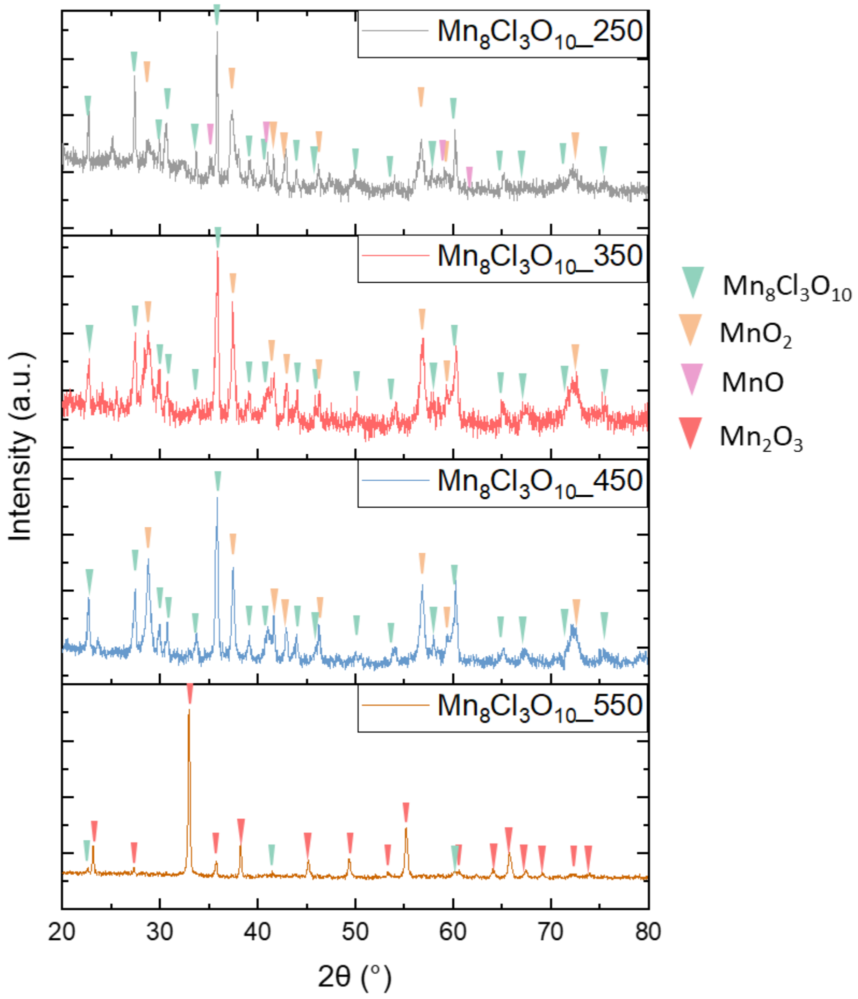

Figure S6: XRD spectra of MnCl-based catalysts synthesized calcined at 250 °C, 350 °C, 450 °C, and 550 °C. The peaks are indexed as $Mn_8Cl_3O_{10}$, MnO_2, MnO, or Mn_2O_3

Table S5: Crystallite size from XRD data by using the Scherrer equation of the MnCl-based catalysts calcined at different temperatures.

Catalyst	$Mn_8Cl_3O_{10}$ crystallite size (nm)	MnO_2 crystallite size (nm)	MnO crystallite size (nm)	Mn_2O_3 crystallite size (nm)
**$Mn_8Cl_3O_{10}$_250**	77.1	23.8	94.9	-
**$Mn_8Cl_3O_{10}$_350**	36.0	20.3	-	-
**$Mn_8Cl_3O_{10}$_450**	63.1	26.9	-	-
**$Mn_8Cl_3O_{10}$_550**	60.5	-	-	87.8

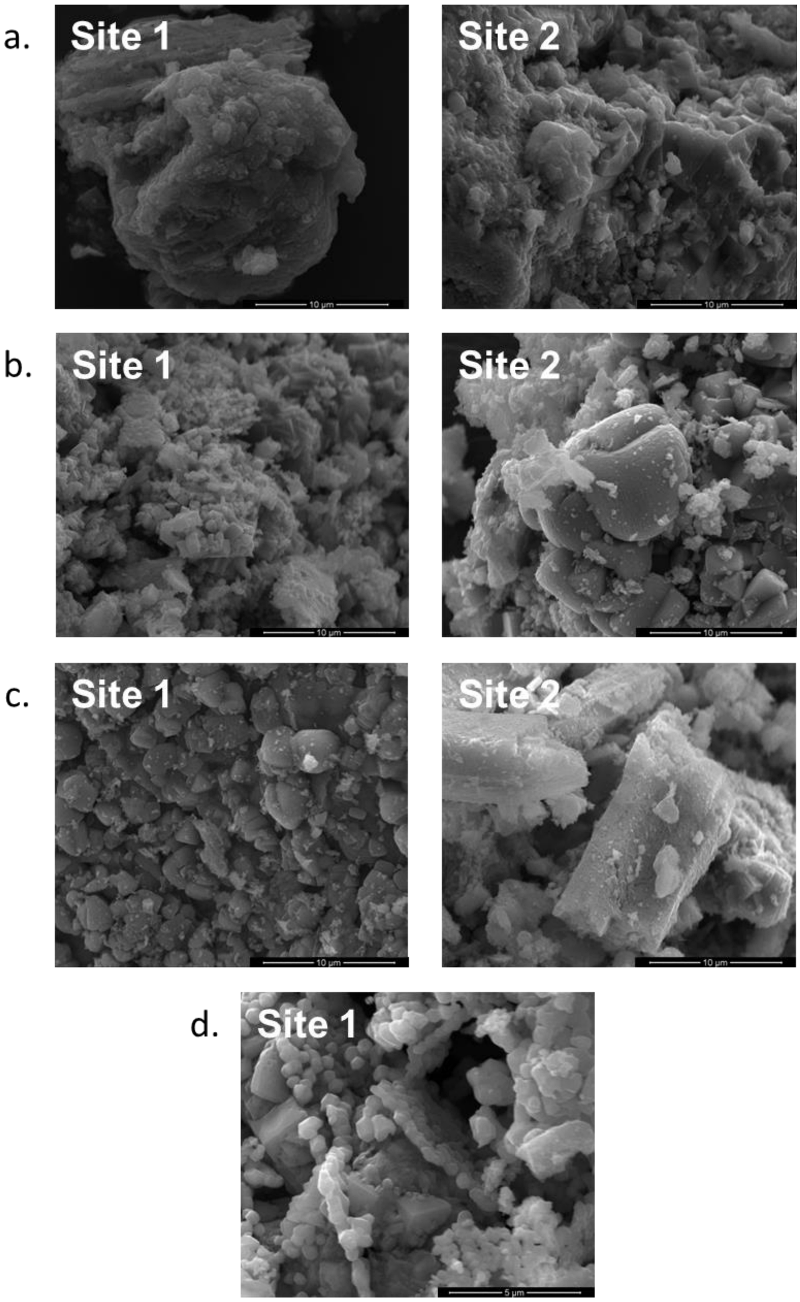

Figure S7: SEM images (magnification 10k) of various sites of the MnCl-based catalysts. a. Mn8Cl3O10 calcined at 250 °C, b. Mn8Cl3O10 calcined at 350 °C, c. Mn8Cl3O10 calcined at 450 °C, and d. site 1 of Mn8Cl3O10 calcined at 550 °C with 20k magnification.

3.3. MnBr-oxides

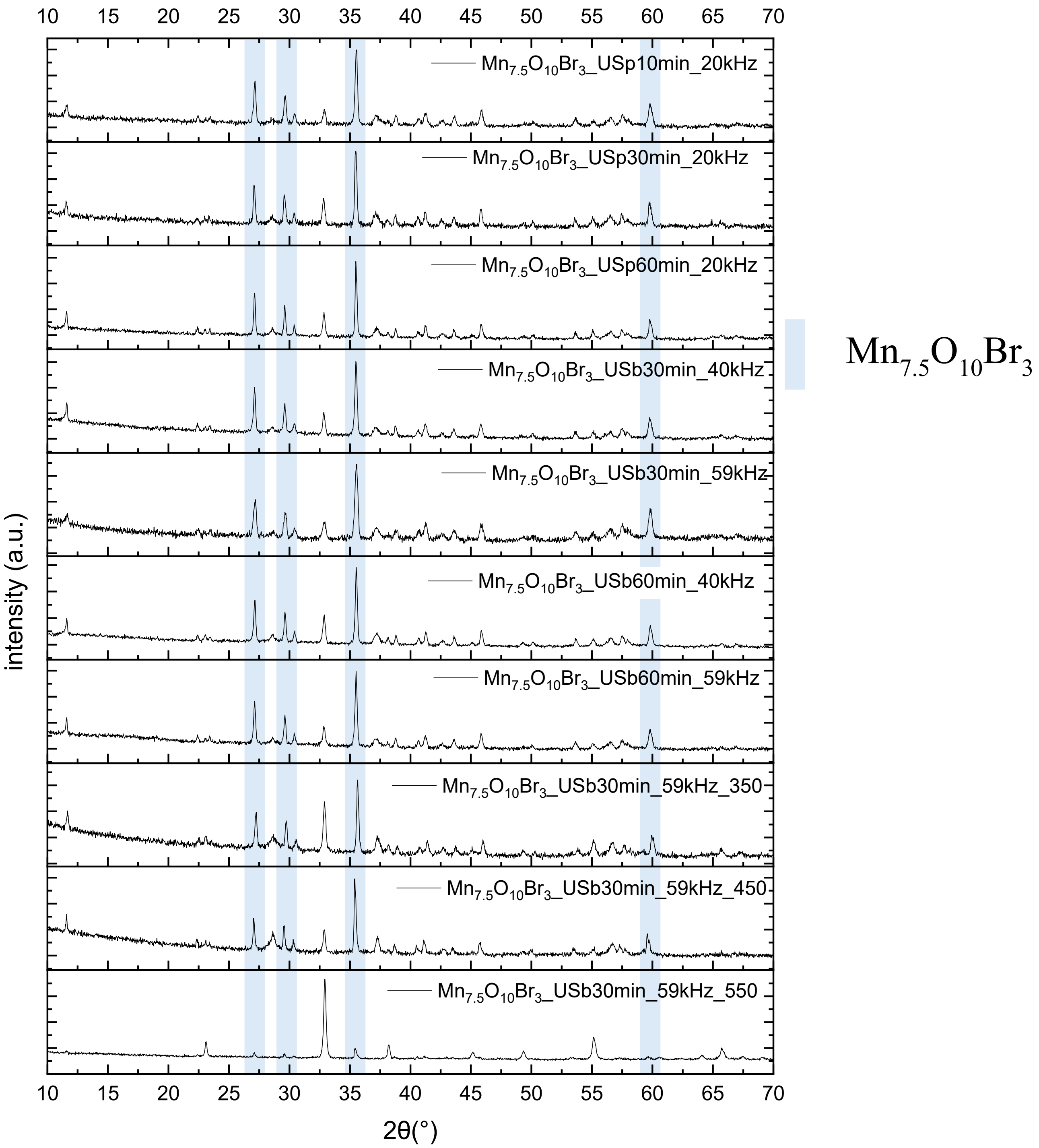

Figure S8: XRD patterns of some Mn7.5O10Br3 samples.

Table S6. *Average crystallite size of* $Mn_{7.5}O_{10}Br_3$.

Samples	D (213) (nm)	D (220) (nm)	D (213) (nm)	Average crystallite size (nm)	Standard Deviation (nm)	Relative error (%)
**$Mn_{7.5}O_{10}Br_3$_250**	122.70	107.80	147.30	125.93	16.29	16.00
**$Mn_{7.5}O_{10}Br_3$_350**	84.00	106.40	105.90	98.77	10.44	11.00
**$Mn_{7.5}O_{10}Br_3$_450**	223.20	106.40	105.50	145.03	55.27	41.00
**$Mn_{7.5}O_{10}Br_3$_250sat**	144.20	178.50	141.90	154.87	16.74	12.00
**$Mn_{7.5}O_{10}Br_3$_250dsat**	106.70	106.10	123.20	112.00	7.92	8.00
**$Mn_{7.5}O_{10}Br_3$_USp10min_20kHz**	66.30	71.70	60.00	66.00	4.78	9.00
**$Mn_{7.5}O_{10}Br_3$_USp30min_20kHz**	87.40	97.30	77.20	87.30	8.21	12.00
**$Mn_{7.5}O_{10}Br_3$_USp60min_20kHz**	216.20	123.80	111.20	150.40	46.81	35.00
**$Mn_{7.5}O_{10}Br_3$_USb30min_40kHz**	72.10	82.50	64.70	73.10	7.30	12.00
**$Mn_{7.5}O_{10}Br_3$_USb60min_40kHz**	44.20	42.10	39.20	41.83	2.05	6.00
**$Mn_{7.5}O_{10}Br_3$_USb30min_59kHz**	115.70	91.80	84.30	97.27	13.39	16.00
**$Mn_{7.5}O_{10}Br_3$_USb60min_59kHz**	97.80	82.50	69.50	83.27	11.57	17.00
**$Mn_{7.5}O_{10}Br_3$_USb30min_59kHz_350**	79.00	71.70	78.00	76.23	3.23	5.00
**$Mn_{7.5}O_{10}Br_3$_USb30min_59kHz_450**	152.10	185.70	141.30	159.70	18.91	14.00
**$Mn_{7.5}O_{10}Br_3$_USb30min_59kHz_550**	128.40	127.70	122.60	126.23	2.58	2.00

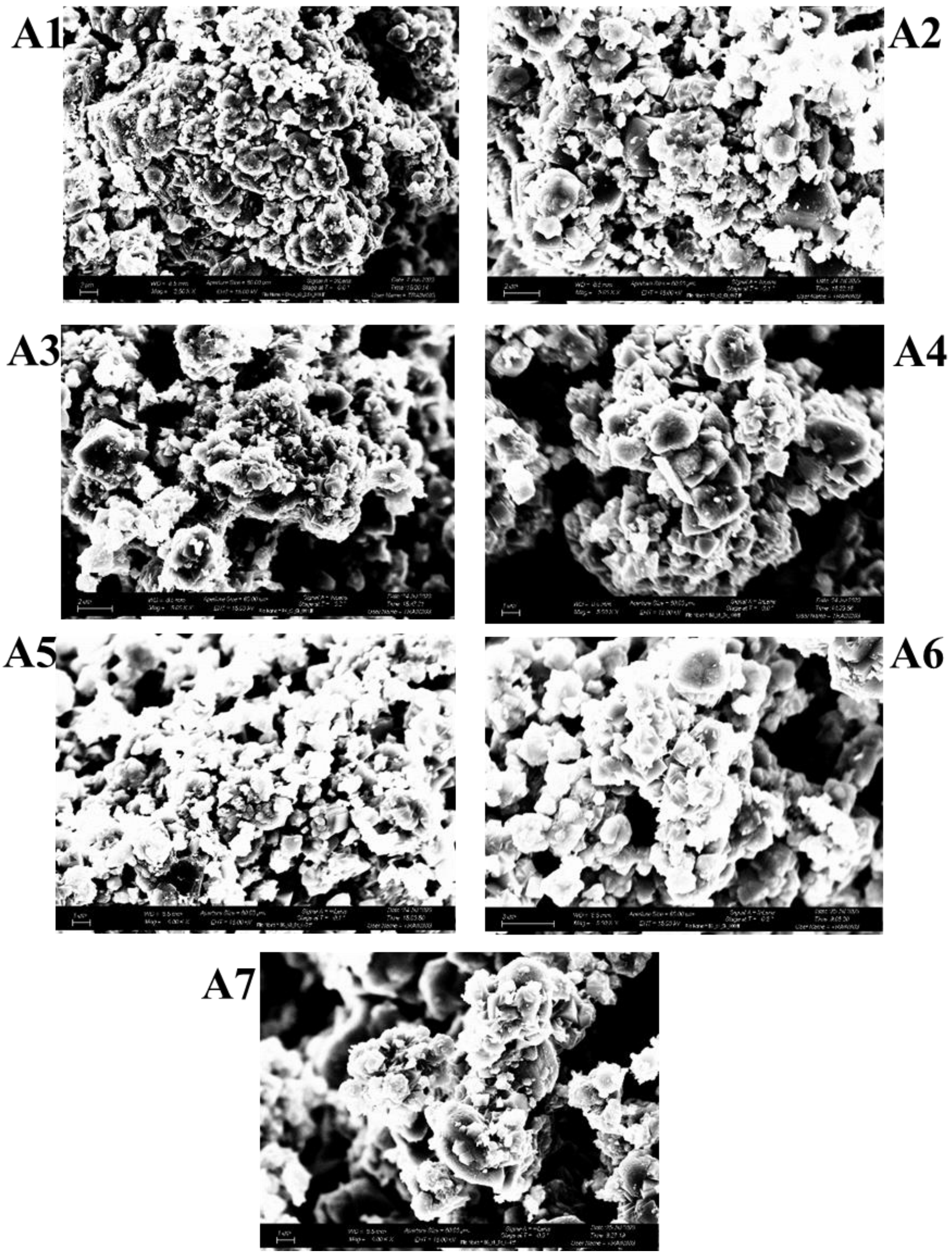

Figure S9: SEM images A1 Mn7.5O10Br3_USp10min_20kHz, A2. Mn7.5O10Br3_USp30min_20kHz, A3. Mn7.5O10Br3_USp60min_20kHz, A4. Mn7.5O10Br3_USb30min_40kHz, A5. Mn7.5O10Br3_USb60min_40kHz, A6. Mn7.5O10Br3_USb30min_59kHz, A7. Mn7.5O10Br3_USb60min_59kHz with magnification

Table S7. *Percentage atomic values of elements (Mn, Br and O) in the samples.*

	**O %at**	**Mn %at**	**Br %at**	**Br:Mn**	**Br:O**	**Mn:O**
**$Mn_{7.5}O_{10}Br_3$_USp10min_20kHz**	52.39	40.81	6.80	0.13	0.17	0.78
**$Mn_{7.5}O_{10}Br_3$_Usp30min_20kHz**	52.93	34.38	12.69	0.37	0.24	0.65
**$Mn_{7.5}O_{10}Br_3$_Usp60min_20kHz**	58.48	31.19	10.36	0.33	0.18	0.53
**$Mn_{7.5}O_{10}Br_3$_Usb30min_40kHz**	55.70	47.77	1.54	0.03	0.03	0.68
**$Mn_{7.5}O_{10}Br_3$_Usb60min_40kHz**	60.11	35.16	4.73	0.13	0.08	0.58
**$Mn_{7.5}O_{10}Br_3$_Usb30min_59kHz**	52.43	36.18	11.38	0.31	0.22	0.69
**$Mn_{7.5}O_{10}Br_3$_Usb60min_59kHz**	61.09	32.58	6.34	0.19	0.10	0.53

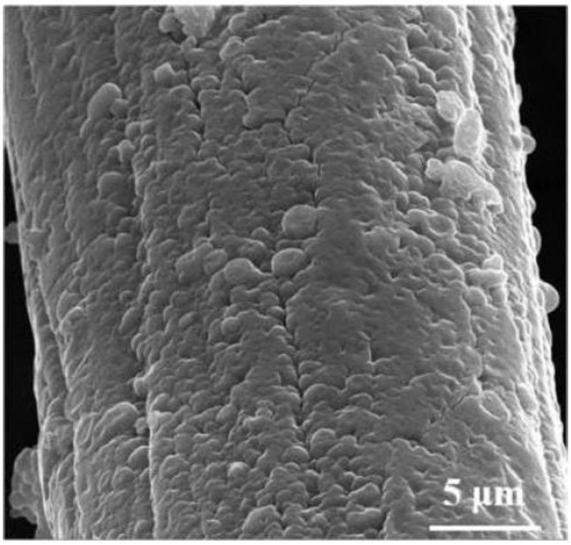

Figure S10: . SEM-EDX images of one site of Mn7.5O10Br3_USb30 min_59kHz_350 sample.

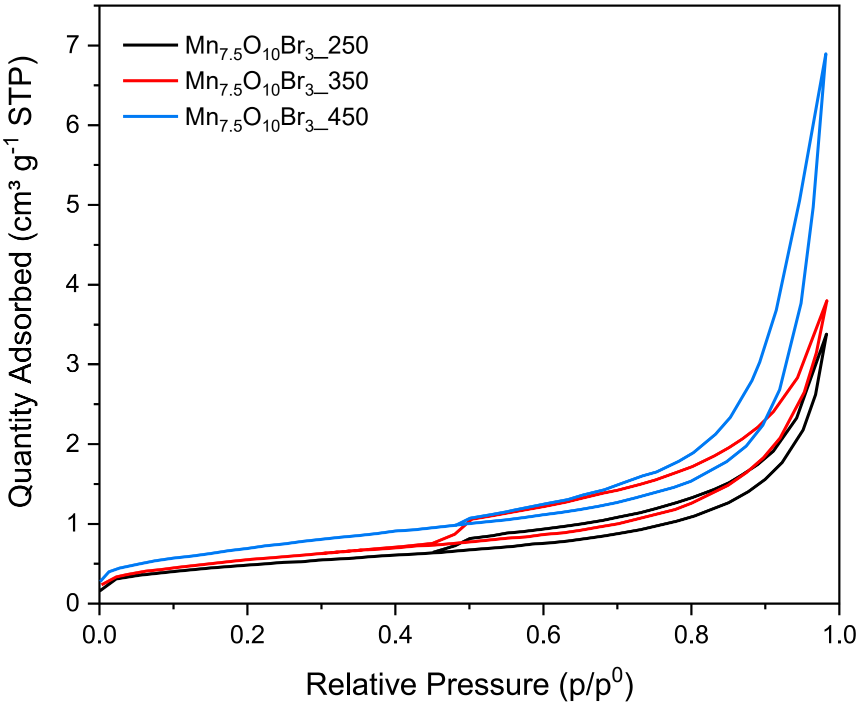

Figure S11: BET analysis of nitrogen adsorption-desorption isotherms for $Mn_{7.5}O_{10}Br_3$ catalysts by varying the calcination T.

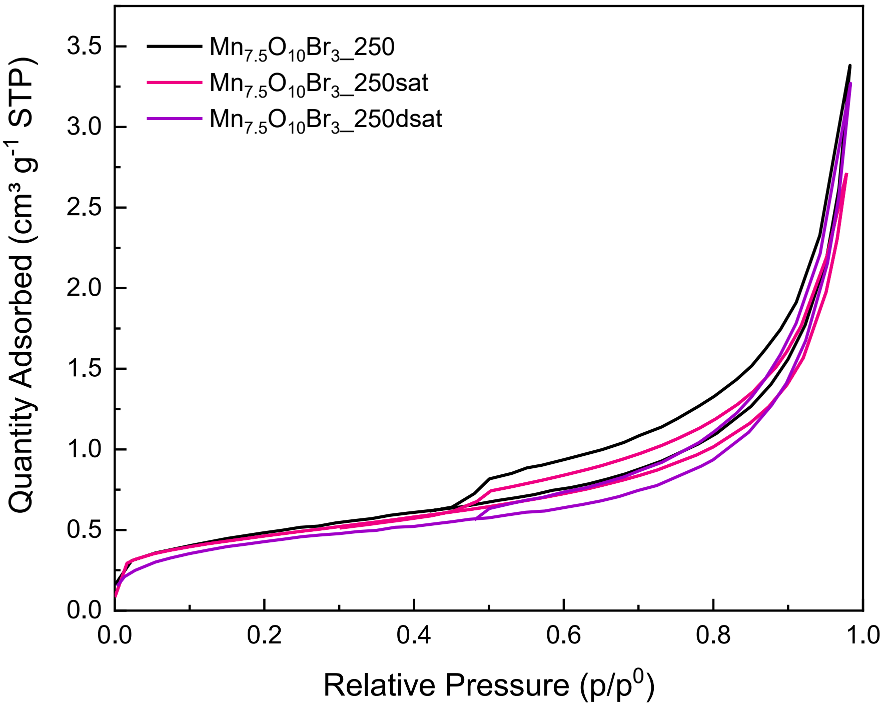

Figure S12: BET analysis of nitrogen adsorption-desorption isotherms for $Mn_{7.5}O_{10}Br_3$ catalysts by varying the precursors' concentration.

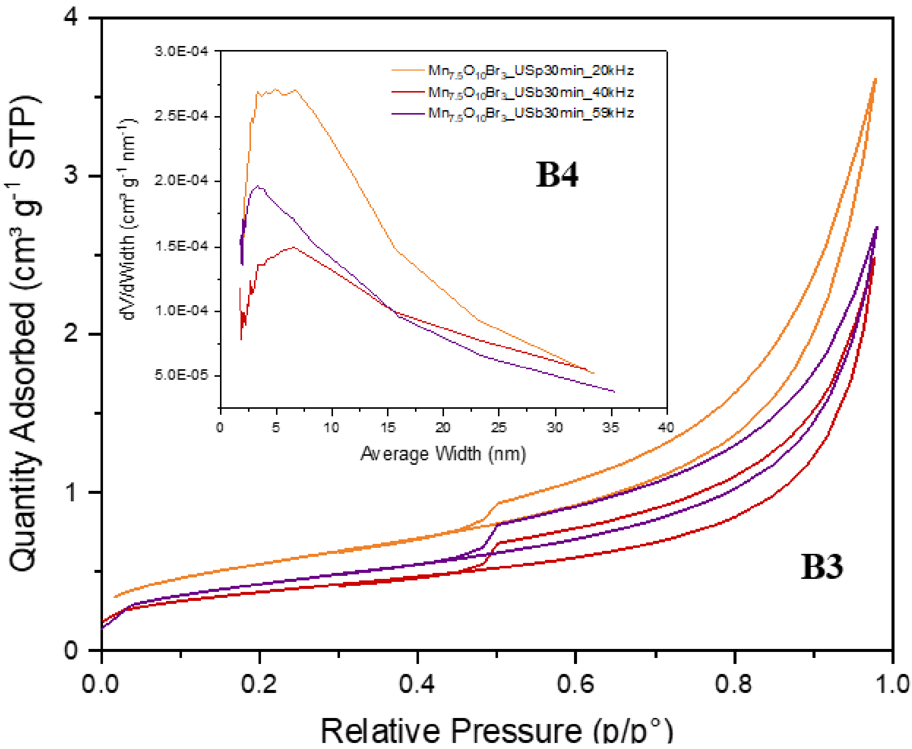

Figure S13: B3. BET analysis of nitrogen adsorption-desorption isotherms and B4. pore size distribution curves (inserted images) by varying USp and USb frequencies at constant sonication t of 30 min.

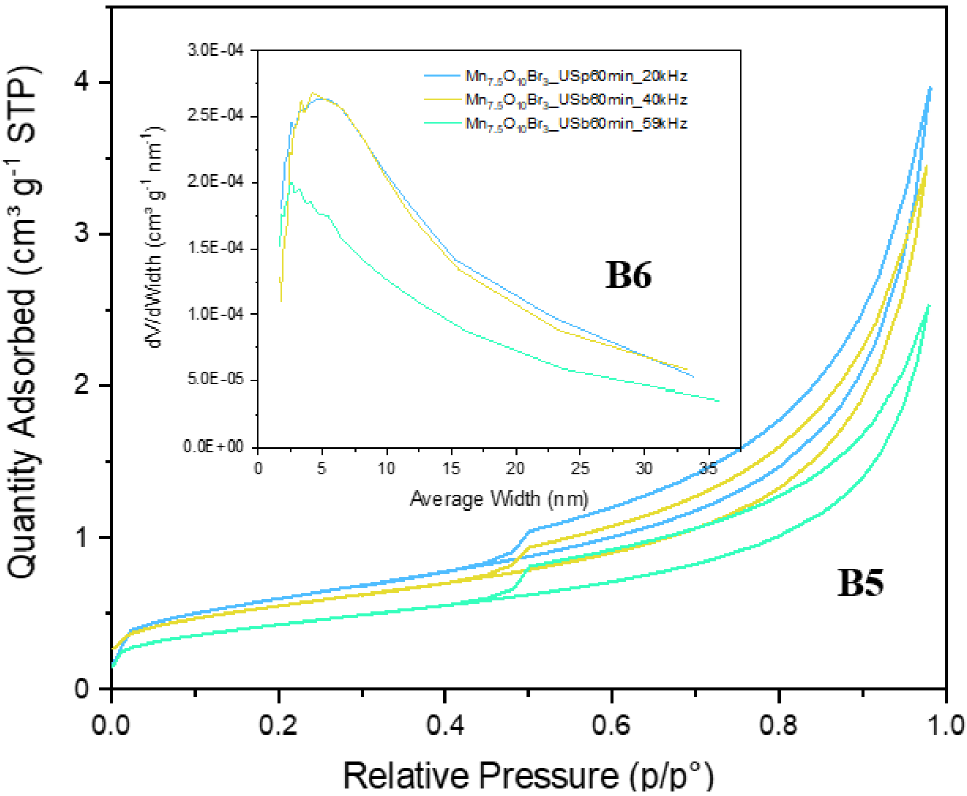

Figure S14: B5. BET analysis of nitrogen adsorption-desorption isotherms and B6. pore size distribution curves (inserted images) by varying USp and USb frequencies at constant sonication t of 60 min.

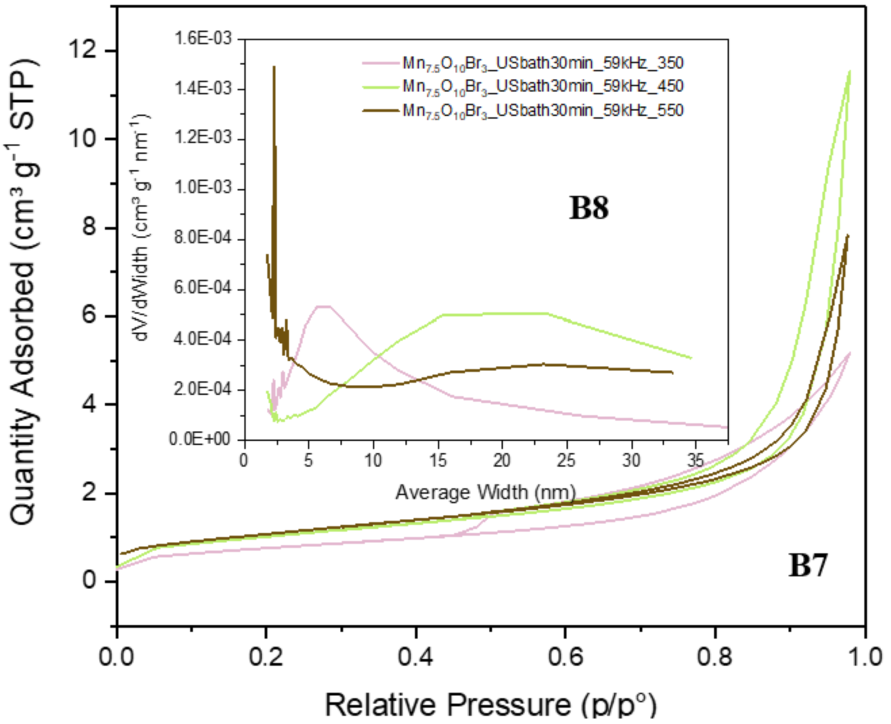

Figure S15: B7. BET analysis of nitrogen adsorption-desorption isotherms and B8. pore size distribution curves (inserted images) by varying calcination T.

Table S8. *BET surface area of all samples.*

Samples	BET surface area ($m^2\,g^{-1}$)
**$Mn_{7.5}O_{10}Br_3$_250**	$1.69 \pm 1.55 \cdot 10^{-2}$
**$Mn_{7.5}O_{10}Br_3$_350**	$1.97 \pm 1.67 \cdot 10^{-2}$
**$Mn_{7.5}O_{10}Br_3$_450**	$2.50 \pm 2.57 \cdot 10^{-2}$
**$Mn_{7.5}O_{10}Br_3$_250sat**	$1.62 \pm 0.88 \cdot 10^{-2}$
**$Mn_{7.5}O_{10}Br_3$_250dsat**	$1.52 \pm 1.51 \cdot 10^{-2}$
**$Mn_{7.5}O_{10}Br_3$_USp10min_20kHz**	$1.57 \pm 0.39 \cdot 10^{-2}$
**$Mn_{7.5}O_{10}Br_3$_USp30min_20kHz**	$2.00 \pm 0.97 \cdot 10^{-2}$
**$Mn_{7.5}O_{10}Br_3$_USp60min_20kHz**	$2.17 \pm 0.10 \cdot 10^{-2}$
**$Mn_{7.5}O_{10}Br_3$_USb30min_40kHz**	$1.33 \pm 0.76 \cdot 10^{-2}$
**$Mn_{7.5}O_{10}Br_3$_USb60min_40kHz**	$1.98 \pm 0.93 \cdot 10^{-2}$
**$Mn_{7.5}O_{10}Br_3$_USb30min_59kHz**	$1.54 \pm 0.60 \cdot 10^{-2}$
**$Mn_{7.5}O_{10}Br_3$_USb60min_59kHz**	$1.57 \pm 0.46 \cdot 10^{-2}$
**$Mn_{7.5}O_{10}Br_3$_USb30min_59kHz_350**	$2.77 \pm 0.11 \cdot 10^{-2}$
**$Mn_{7.5}O_{10}Br_3$_USb30min_59kHz_450**	$3.70 \pm 1.76 \cdot 10^{-2}$
**$Mn_{7.5}O_{10}Br_3$_USb30min_59kHz_550**	$3.91 \pm 0.55 \cdot 10^{-2}$

3.3.1. Principal Component Analysis (PCA)

A PCA has been conducted after obtaining all the discrete data, such as $Mn_{7.5}O_{10}Br_3$ phase quantification through XRD analysis, BET surface area, crystallite size, overpotential, and Tafel slope.

Principal Component Analysis (PCA) is a technique used to simplify datasets by reducing the number of variables while preserving the most relevant information. It identifies new axes, called Principal Components, that capture the directions of the most significant variance in the data. Each sample is projected onto these components.

The first components usually explain most of the meaningful structure, while the remaining ones mostly describe noise. In this case, the dataset's dimension was insufficient, so all components were investigated (see Figure S16). In the end, only the first two of them turned out to be meaningful.

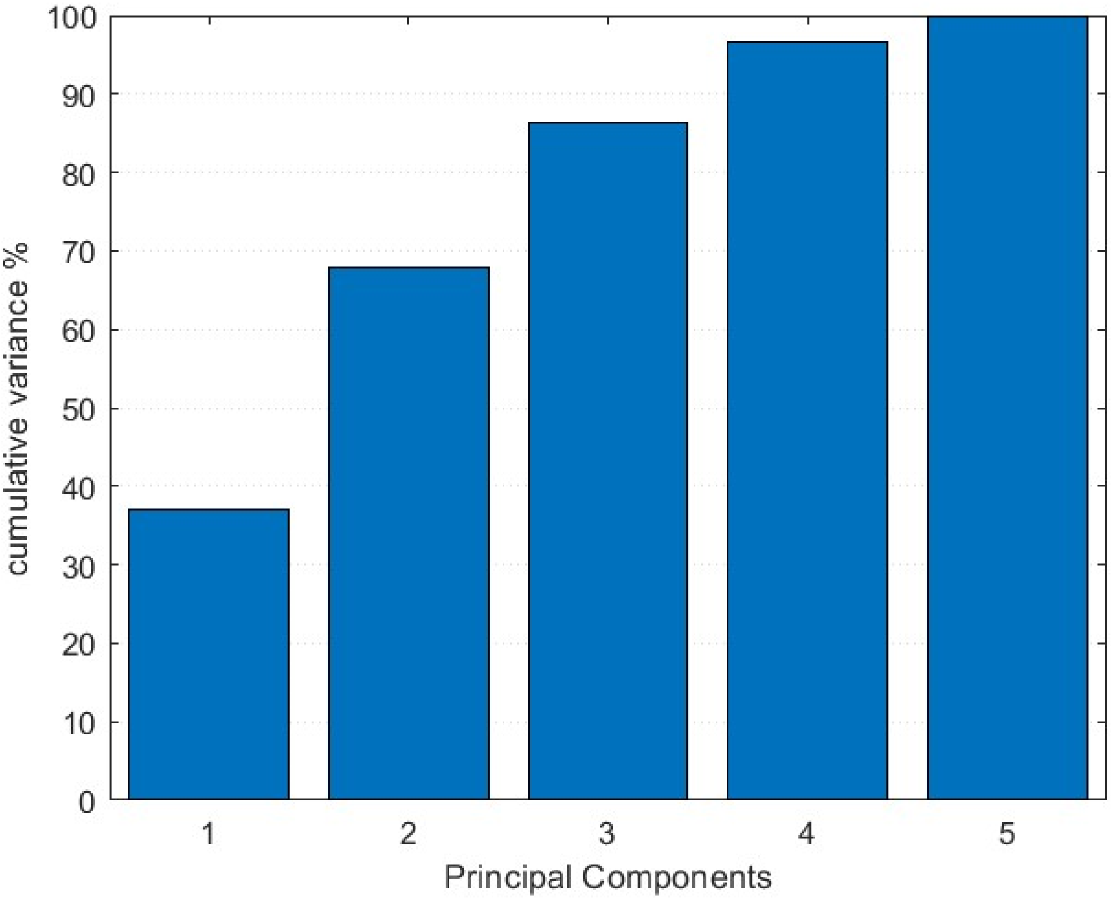

Figure S16: Cumulative variance depending on the number of principal components considered.

Autoscaling the data ensures all variables contribute equally by centering and normalizing them. This passage is crucial, because the chosen variables (XRD quantification, BET surface area, overpotential and Tafel slope) are very different in terms of order of magnitude but not in terms of significance. The discreet data have been preprocessed to align them at the same level and with the same importance for the model. Autoscale has been used as a data scaling technique prior to the calculations.

By using class labels related to the variables used during the synthesis (homogenization source, time, calcination T, Br-precursor amount), it is possible to detect variation in the principal component due to different classes. In Figure S17 it is possible to see that on direction 2 the samples are divided into two big groups: low amount of Br-precursor (4.8 and 5 moles) and high amount (10 moles). There is one outlier, but the model is generally good enough. The plot with the residuals is reported in Figure S18, all the samples are very well modeled, except for one (higher Q residual).

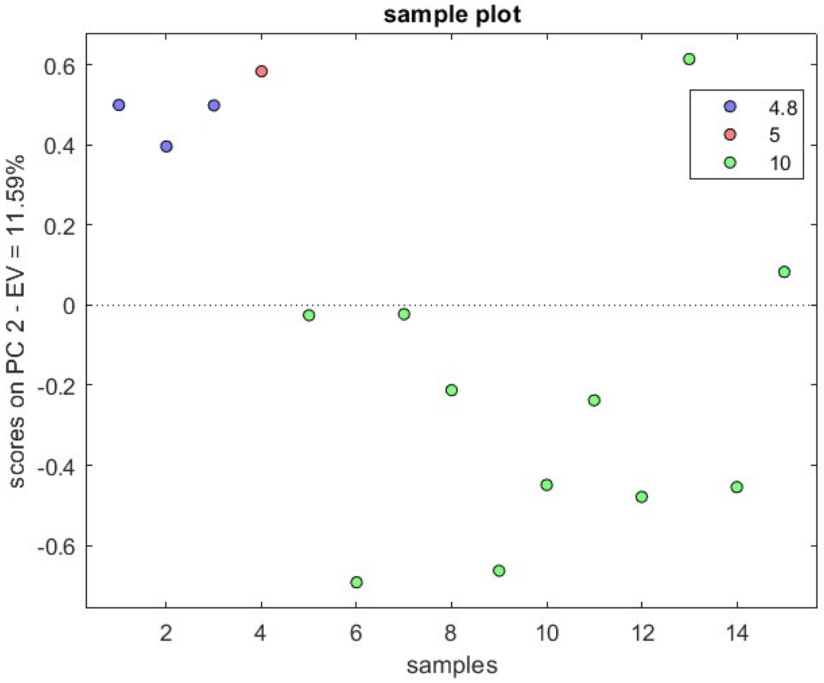

Figure S17: Effect of Br-precursor amount (in moles, 4.8, 5 and 10 moles). The axes represent the samples (in order as presented in the work) vs principal component 2.

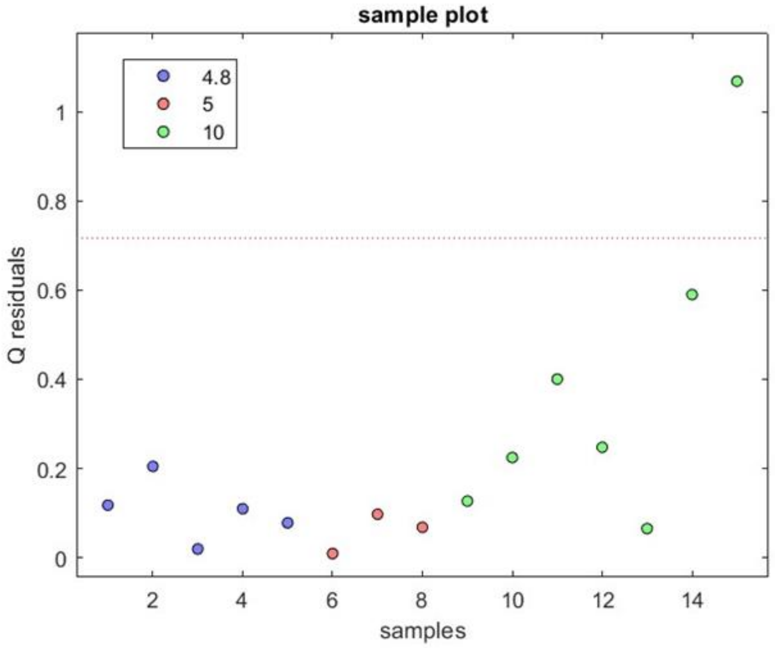

Figure S18: Effect of Br-precursor amount (4.8, 5, and 10 moles) on Q residuals. Blue, red, and green points correspond to 4.8, 5, and 10 moles of Br-precursor, respectively.

3.4. Electrochemical characterization

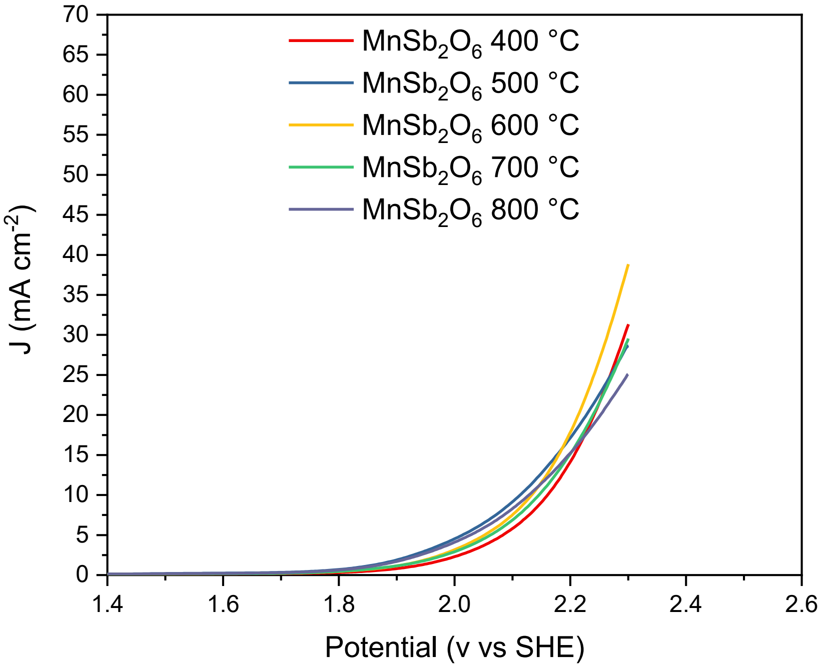

Figure S19: RDE test, LSV1 curves of MnSb-based catalysts calcined at different temperatures. Catalyst loading 0.75 mg cm-2, reference electrode Ag/AgCl, counter electrode Pt wire. Potential range 0-2.3 V vs SHE. Tested in 0.5 M H_2SO_4.

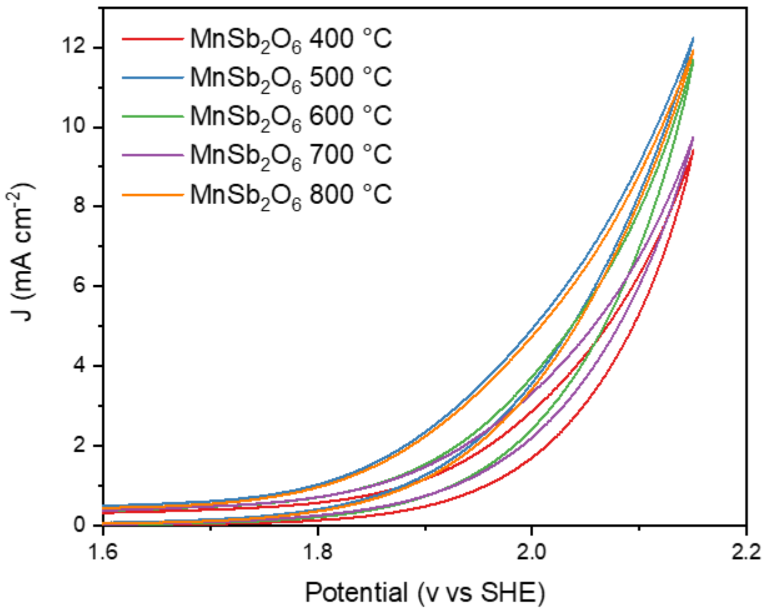

Figure S20: RDE test, CV curves of MnSb-based catalysts calcined at different temperatures. Catalyst loading 0.75 mg cm^{-2}, reference electrode Ag/AgCl, counter electrode Pt wire. Potential range 0-2.15 V vs SHE. Magnification from 1.6 to 2.2 V. Tested in 0.5 M H_2SO_4.

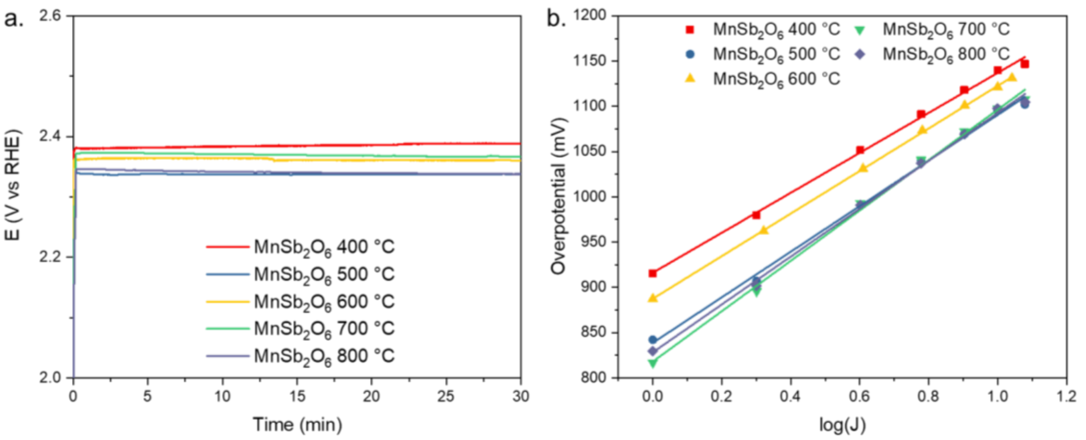

Figure S21: RDE test, a. CP curves of MnSb-based catalysts calcined at different temperatures and 10 mA cm^{-2}, b. Tafel plots of MnSb-based catalysts calcined at different temperatures. Catalyst loading: 0.75 mg cm^{-2}, reference electrode Ag/AgCl, counter electrode Pt wire. Tested in 0.5 M H_2SO_4.

Table S9: RDE test, overpotential at 10 mA cm^{-2} and Tafel slope values of MnSb-based catalysts calcined at different temperatures. Tested in 0.5 M H_2SO_4.

Catalyst	Overpotential, η (mV, at 10 mA cm^{-2})	Tafel Slope (mV dec^{-1})
$MnSb_2O_6$_400	1148	220
$MnSb_2O_6$_500	1072	251
$MnSb_2O_6$_600	1121	235
$MnSb_2O_6$_700	1131	276
$MnSb_2O_6$_800	1072	265

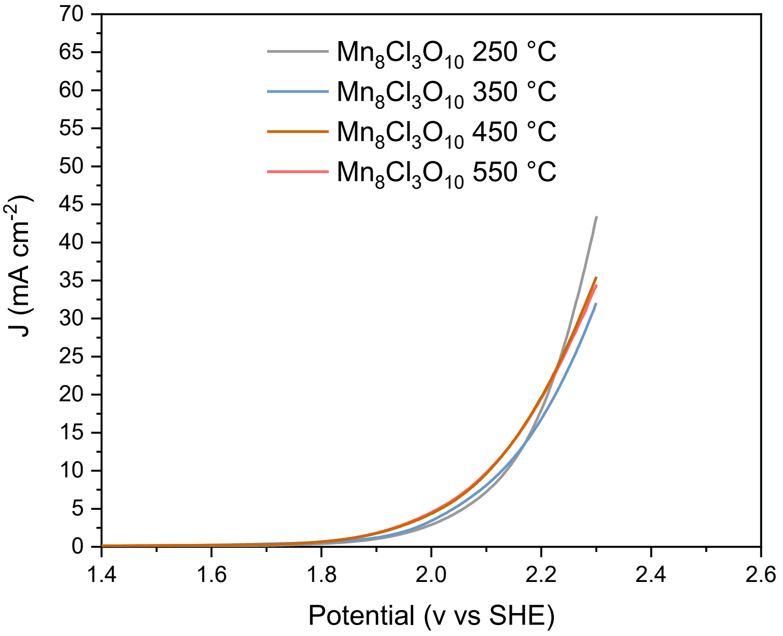

Figure S22: RDE test, LSV1 curves of MnCl-based catalysts calcined at different temperatures. Catalyst loading: 0.75 mg cm^{-2}, reference electrode Ag/AgCl, counter electrode Pt wire. Potential range 0-2.3 V vs SHE. Tested in 0.5 M H_2SO_4.

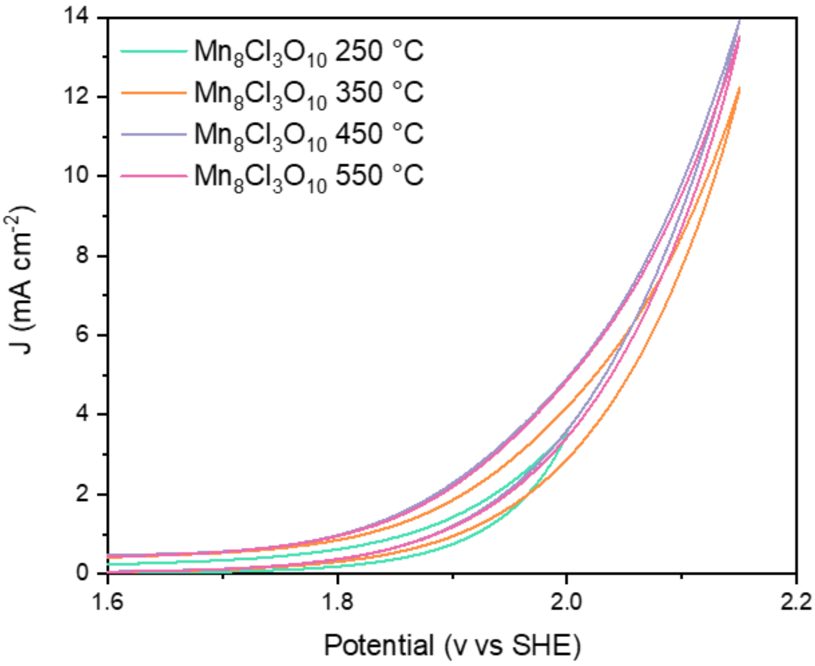

Figure S23: RDE test, CV curves of MnCl-based catalysts calcined at different temperatures. Catalyst loading: 0.75 mg cm^{-2}, reference electrode Ag/AgCl, counter electrode Pt wire. Potential range 0-2.15 V vs SHE. Magnification from 1.6 to 2.2 V vs SHE. Tested in 0.5 M H_2SO_4.

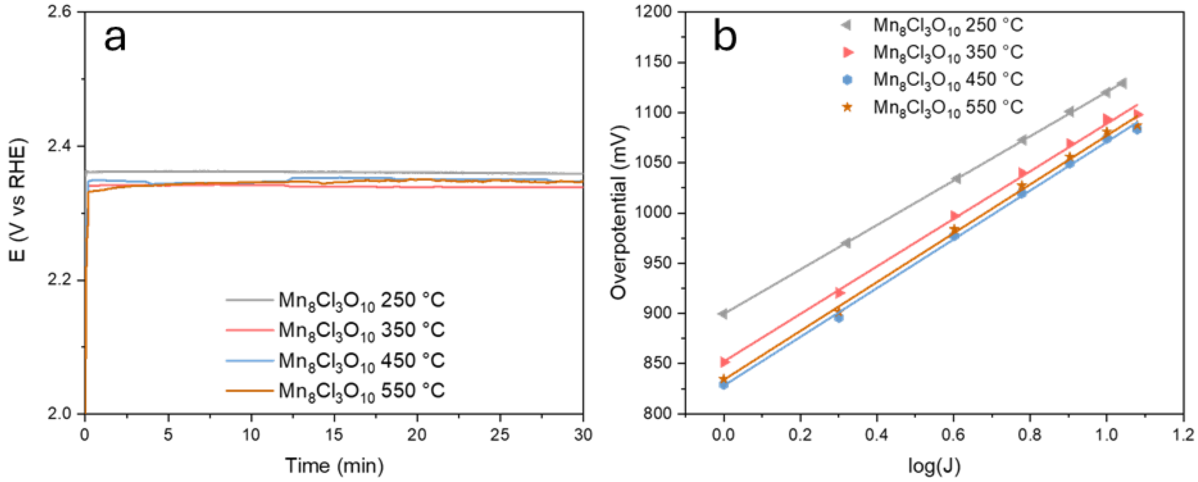

Figure S24: RDE test, a. CP curves of MnCl-based catalysts calcined at different temperatures and 10 mA cm^{-2}, b. Tafel plots of MnSb-based catalysts calcined at different temperatures. Catalyst loading: 0.75 mg cm^{-2}, reference electrode Ag/AgCl, counter electrode Pt wire. Potential range 0-2.15 V vs SHE. Tested in 0.5 M H_2SO_4.

Table S10: RDE test, overpotential at 10 mA cm^{-2} and Tafel slope values of MnSb-based catalysts calcined at different temperatures. Tested in 0.5 M H_2SO_4.

**Catalyst**	**Overpotential, η (mV, at 10 mA cm^{-2})**	**Tafel Slope (mV dec^{-1})**
**$Mn_8Cl_3O_{10}$_250**	1120	221
**$Mn_8Cl_3O_{10}$_350**	1081	237
**$Mn_8Cl_3O_{10}$_450**	1084	243
**$Mn_8Cl_3O_{10}$_550**	1086	243

Firstly, a comparison between the samples obtained with the highest amount of Br-precursor calcined at three different temperatures have been compared (see **Figure S25**, **Figure S26**, and **Figure S27**).

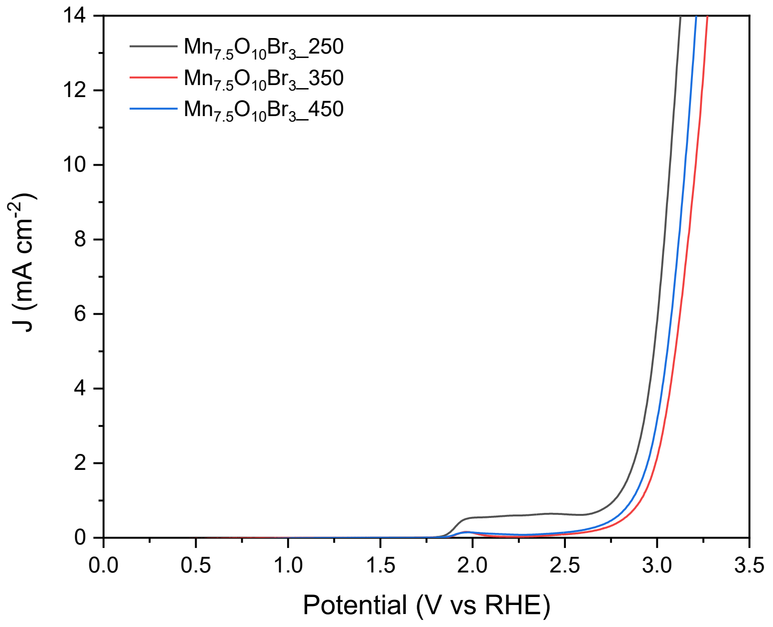

Figure S25. *LSV curves for $Mn_{7.5}O_{10}Br_3$ calcined at 250 °C, 350 °C and 450 °C at 5 mV s^{-1} in 0.5 M H_2SO_4. Tested in 0.5 M H_2SO_4.*

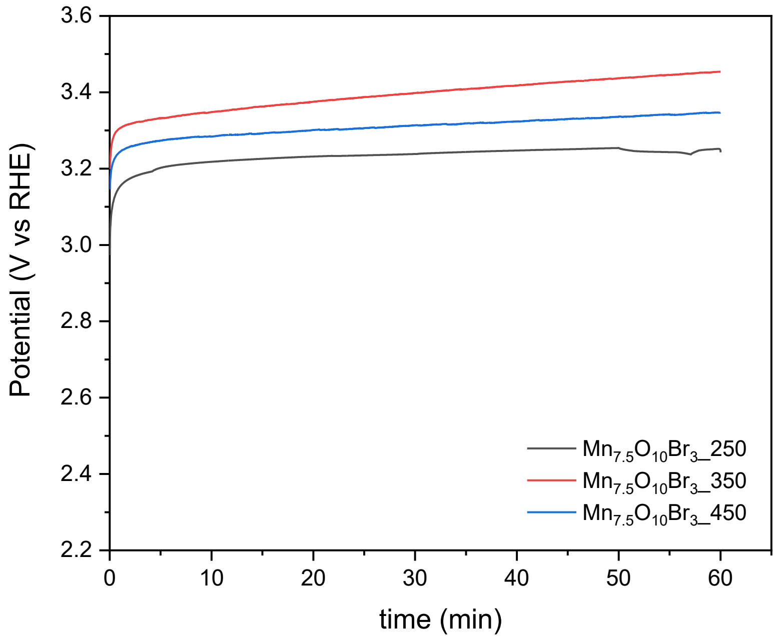

Figure S26. *CP curves for $Mn_{7.5}O_{10}Br_3$ calcined at 250 °C, 350 °C and 450 °C at 10 mA cm^{-2} in 0.5 M H_2SO_4. Tested in 0.5 M H_2SO_4.*

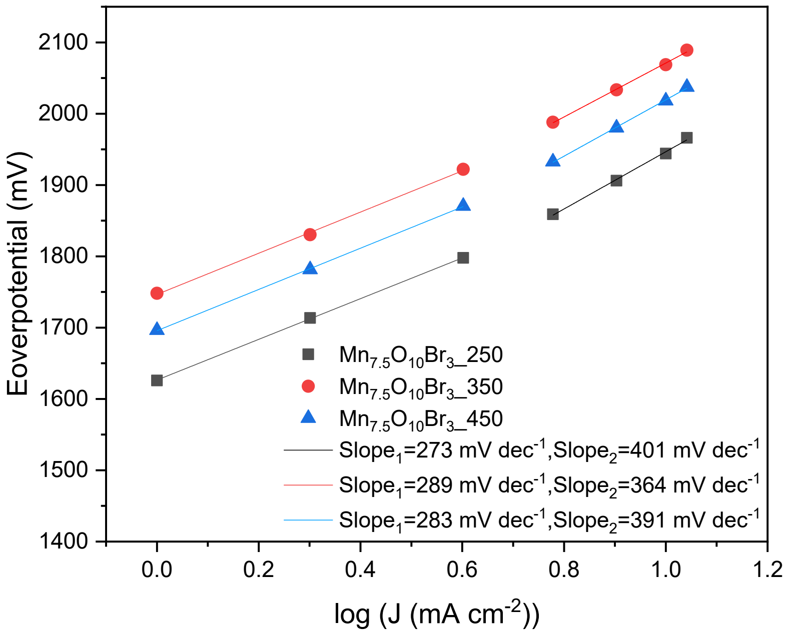

Figure S27. *Tafel plots for $Mn_{7.5}O_{10}Br_3$ calcined at 250 °C, 350 °C and 450 °C in 0.5 M H_2SO_4.*

Subsequently, the comparison is performed by changing the $MnBr_2 \cdot 4H_2O$ precursor concentration from 3mL x 1.6M, 1mL x 5M, to 2mL x 5M. to 5 M. These samples are called $Mn_{7.5}O_{10}Br_3$_250 (black line),

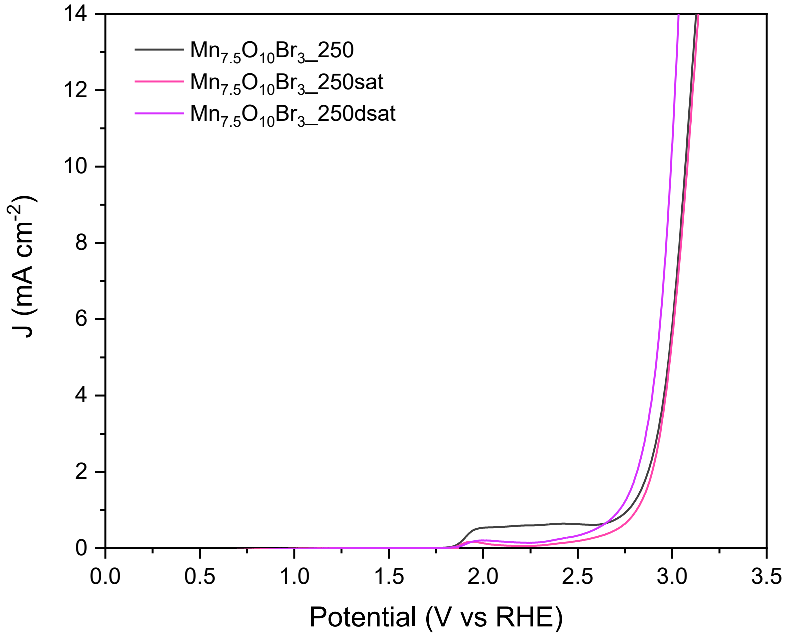

Figure S28. *LSV curves for $Mn_{7.5}O_{10}Br_3$_250, $Mn_{7.5}O_{10}Br_3$_250sat and $Mn_{7.5}O_{10}Br_3$_250dsat at 5 mV s^{-1}. Tested in 0.5 M H_2SO_4.*

$Mn_{7.5}O_{10}Br_3$_250sat (pink line) and $Mn_{7.5}O_{10}Br_3$_250dsat (purple line), respectively (see **Figure S28** and **Figure S29**))

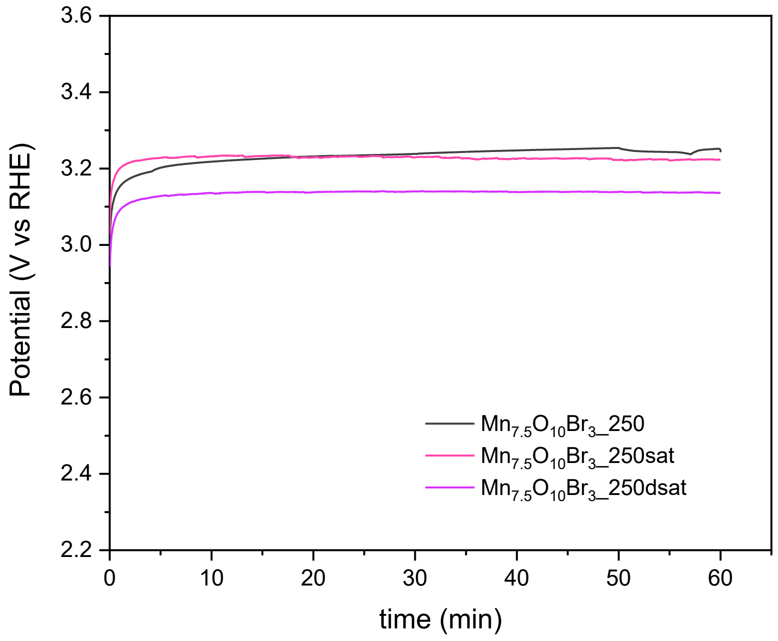

Figure S29. *CP curves for $Mn_{7.5}O_{10}Br_3$_250, $Mn_{7.5}O_{10}Br_3$_250sat and $Mn_{7.5}O_{10}Br_3$_250dsat samples at 10 mA cm^{-2}. Tested in 0.5 M H_2SO_4.*

Finally, the Tafel slope plots are reported in **Figure S30.** The values of the Tafel slope are reported for low values of current density (1, 2, and 4 mA cm^{-2}) and high current density values (6, 8, 10, and 11 mA cm^{-2}). It is to be highlighted that the behaviour at higher current densities is more relevant for the practical application of these catalysts that needs to be used at currents of the order of amperes.

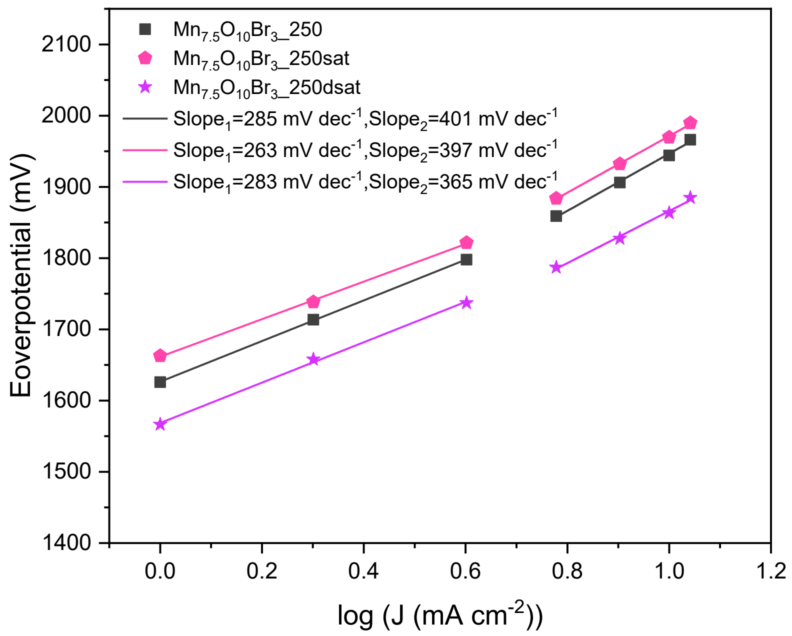

Figure S30. *Tafel Plots for $Mn_{7.5}O_{10}Br_3$_250, $Mn_{7.5}O_{10}Br_3$_250sat and $Mn_{7.5}O_{10}Br_3$_250dsat. Tested in 0.5 M H_2SO_4.*

The results of XRD characterizations combined with the outcomings from electrochemical tests highlighted the $Mn_{7.5}O_{10}Br_3$_250dsat as the best sample. Subsequently, the optimization of the mixing step was carried out and the effects have been investigated through physical characterization and electrochemical tests. Firstly, the effect of the variation of the sonication time of the US probe. The frequency is fixed at 20 kHz for the US probe. The time goes from 10 min, 30 min, up to 60 min. The LSV, CVs curves and Tafel slops are shown in the **Figure S31**, **Figure S32**, and **Figure S33**, respectively.

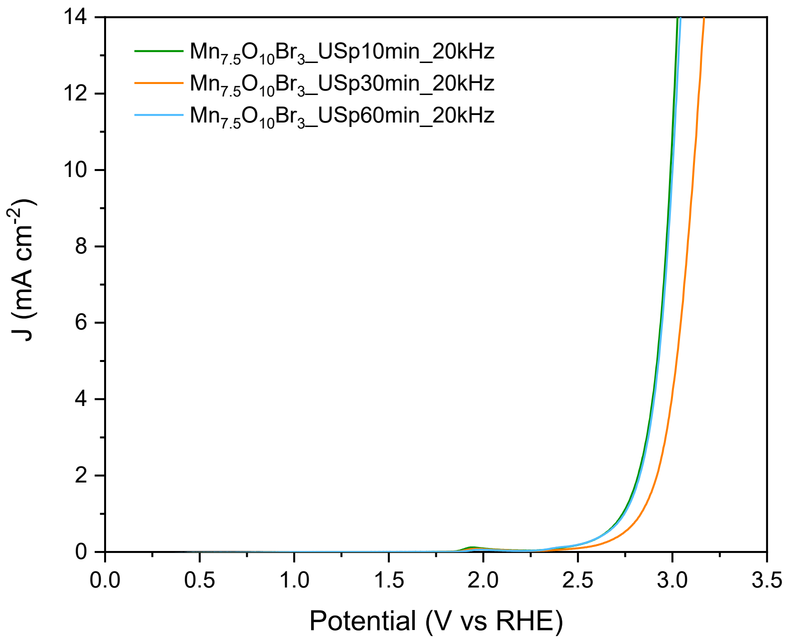

Figure S31. *LSV curves for $Mn_{7.5}O_{10}Br_3$_USp10min_20kHz, $Mn_{7.5}O_{10}Br_3$_USp30min_20kHz and $Mn_{7.5}O_{10}Br_3$_USp60min_20kHz samples at 5 mV s^{-1}. Tested in 0.5 M H_2SO_4.*

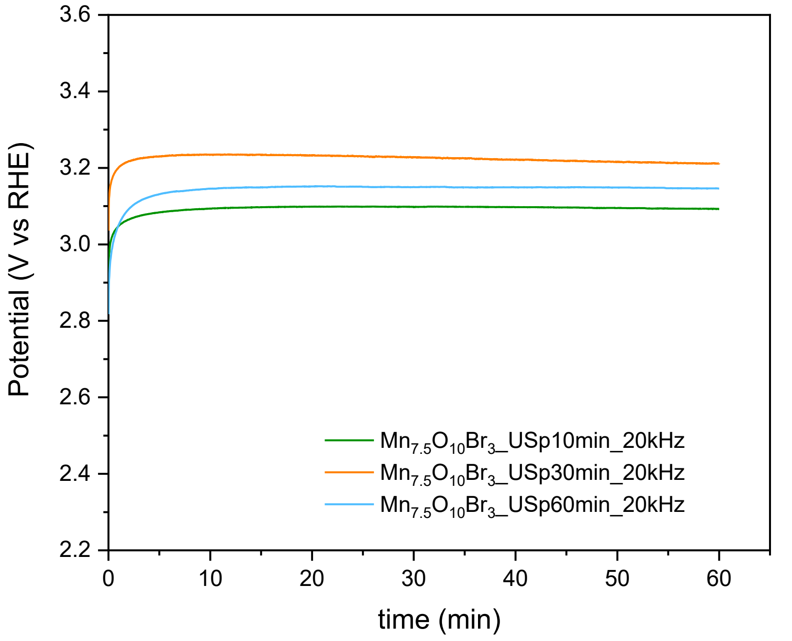

Figure S32. *CP curves for $Mn_{7.5}O_{10}Br_3$_USp10min_20kHz, $Mn_{7.5}O_{10}Br_3$_USp30min_20kHz and $Mn_{7.5}O_{10}Br_3$_USp60min_20kHz samples at 10 mA cm^{-2}. Tested in 0.5 M H_2SO_4.*

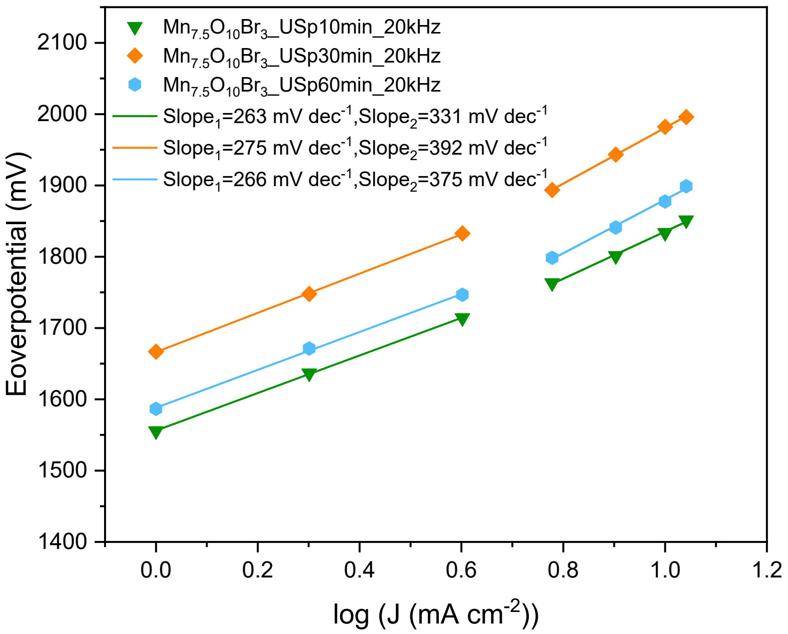

Figure S33. *Tafel Plots for $Mn_{7.5}O_{10}Br_3$_USp10min_20kHz, $Mn_{7.5}O_{10}Br_3$_USp30min_20kHz and $Mn_{7.5}O_{10}Br_3$_USp60min_20kHz. Tested in 0.5 M H_2SO_4.*

Subsequently, the samples homogenized in the US bath with a fixed sonication time of 30 min, but varying the frequency, are compared. The electrocatalytic performances are evaluated via LSV curves, CP curves and Tafel slope values and the results are reported in **Figure S34-Figure S36**.

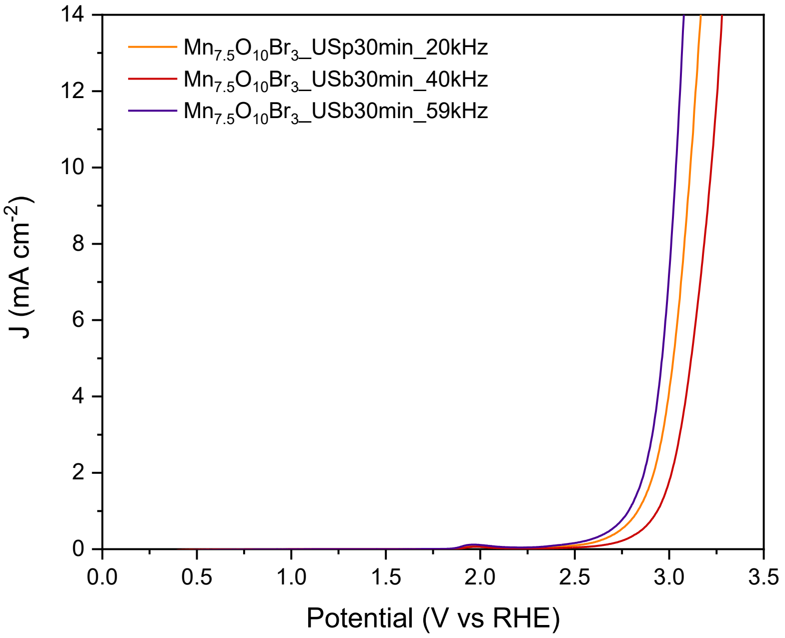

Figure S34*. LSV curves for $Mn_{7.5}O_{10}Br_3$_USp30min_20kHz, $Mn_{7.5}O_{10}Br_3$_USp30min_40kHz and $Mn_{7.5}O_{10}Br_3$_USp30min_59kHz at 5 mV s^{-1}. Tested in 0.5 M H_2SO_4.*

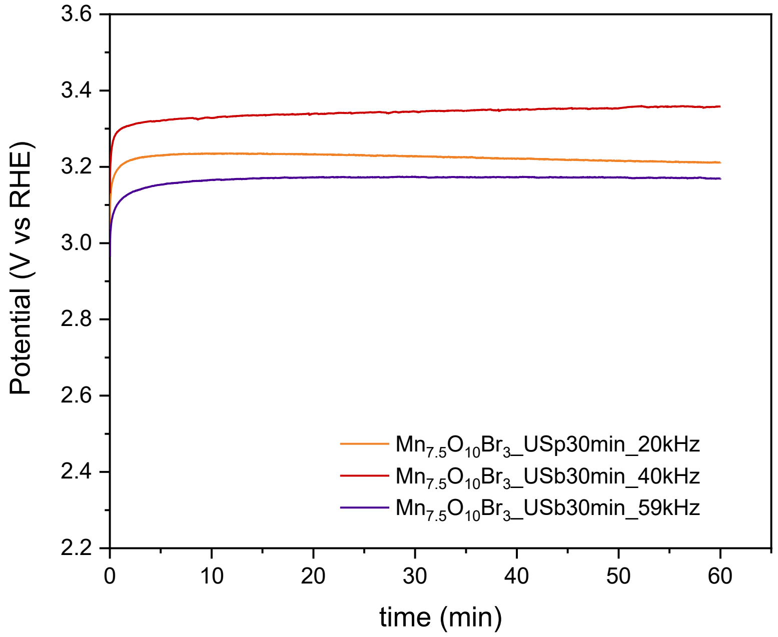

Figure S35. *CP curves for $Mn_{7.5}O_{10}Br_3$_USp30min_20kHz, $Mn_{7.5}O_{10}Br_3$_USb30min_40kHz and $Mn_{7.5}O_{10}Br_3$_USb30min_59kHz samples at 10 mA cm^{-2}. Tested in 0.5 M H_2SO_4.*

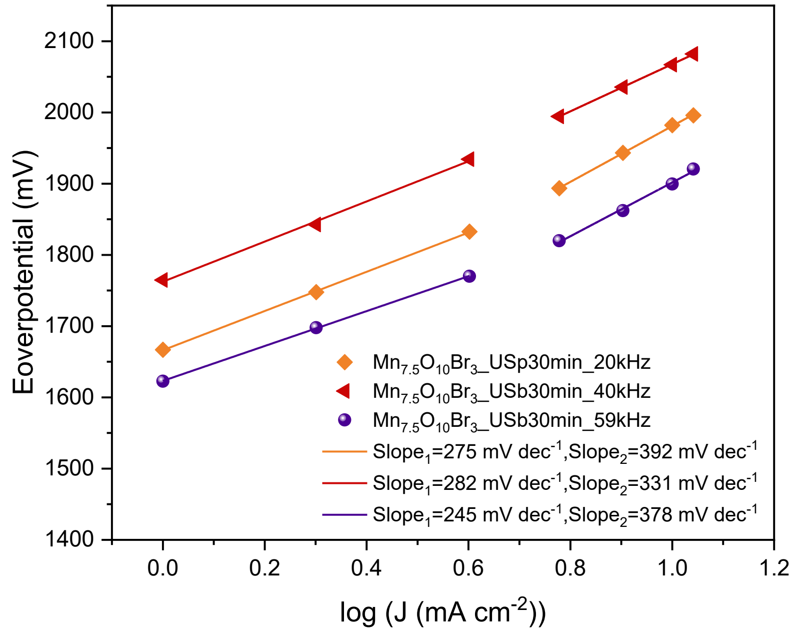

Figure S36. *Tafel Plots for $Mn_{7.5}O_{10}Br_3$_USp30min_20kHz, $Mn_{7.5}O_{10}Br_3$_USp30min_40kHz and $Mn_{7.5}O_{10}Br_3$_USp30min_59kHz. Tested in 0.5 M H_2SO_4.*

A comparison like the previous one is made for the $Mn_{7.5}O_{10}Br_3$_USp60min_20kHz, $Mn_{7.5}O_{10}Br_3$_USb60min_40kHz and $Mn_{7.5}O_{10}Br_3$_USb60min_59kHz samples, obtained by varying the frequency (20 kHz, 40 kHz and 59 kHz), but keeping the sonication time fixed at 60 min. The first sample ($Mn_{7.5}O_{10}Br_3$_USp60min_20kHz) was obtained using by US source an US probe, for the other it was an US bath. (**Figure S38**, **Figure S37**, **Figure S39**)

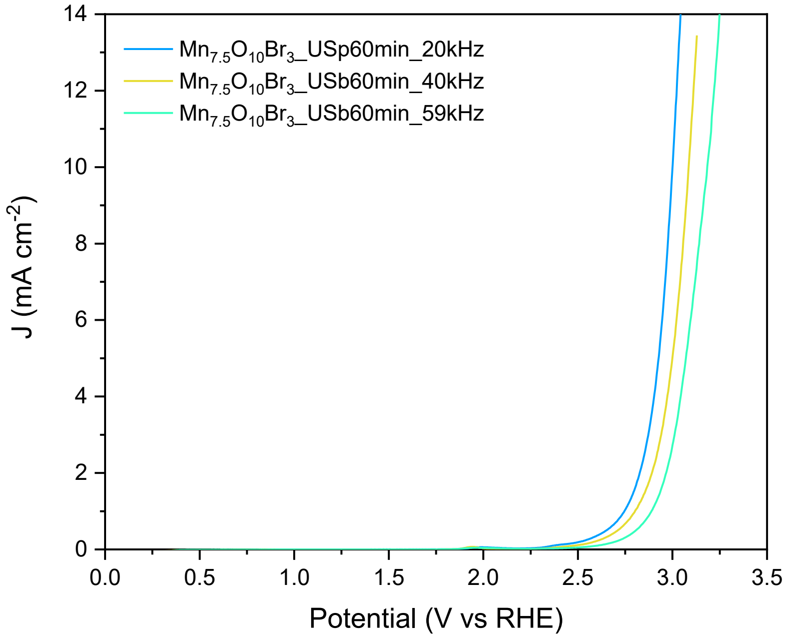

Figure S38. *LSV curves for $Mn_{7.5}O_{10}Br_3$_USp60min_20kHz, $Mn_{7.5}O_{10}Br_3$_USp60min_40kHz and $Mn_{7.5}O_{10}Br_3$_USp60min_59kHz at 5 mV s^{-1}. Tested in 0.5 M H_2SO_4.*

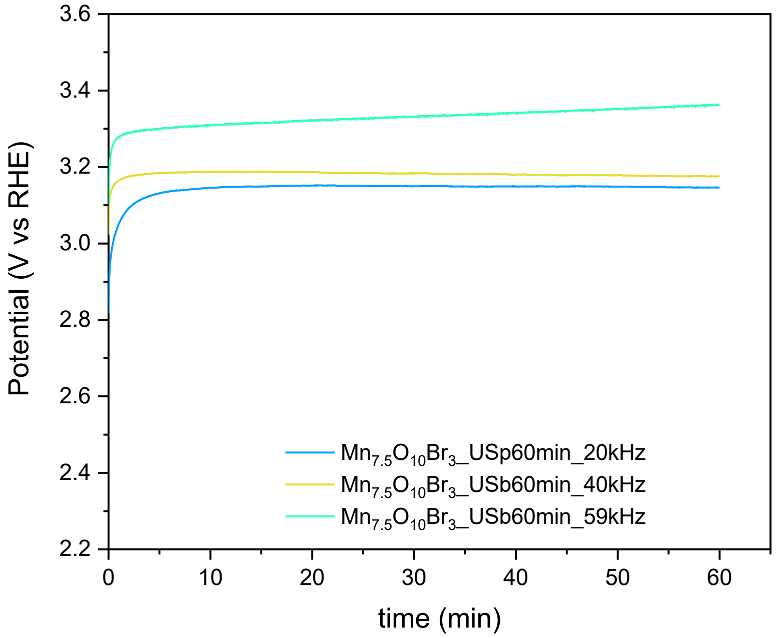

Figure S37*. CP curves for $Mn_{7.5}O_{10}Br_3$_USp60min_20kHz, $Mn_{7.5}O_{10}Br_3$_USb60min_40kHz and $Mn_{7.5}O_{10}Br_3$_USb60min_59kHz samples at 10 mA cm^{-2}. Tested in 0.5 M H_2SO_4.*

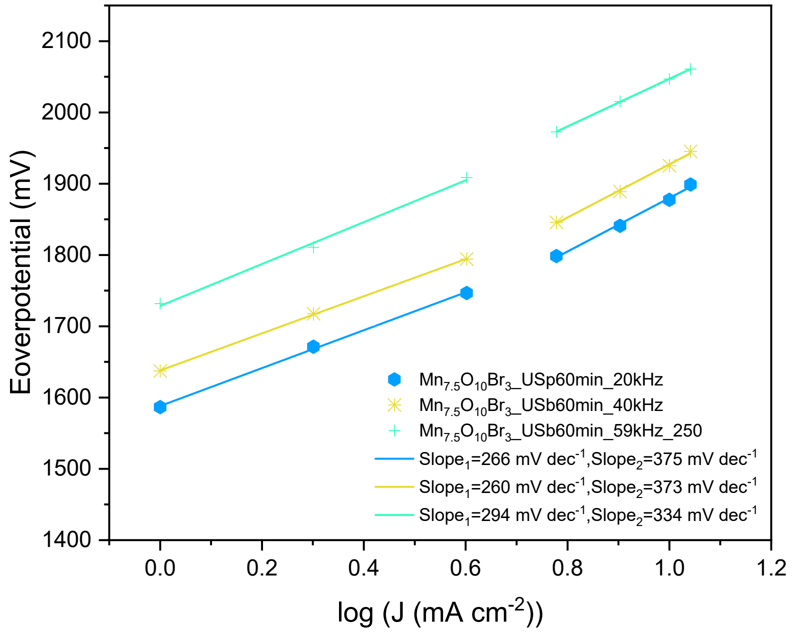

Figure S39. *Tafel Plots for $Mn_{7.5}O_{10}Br_3$_USp60min_20kHz, $Mn_{7.5}O_{10}Br_3$_USb60min_40kHz and $Mn_{7.5}O_{10}Br_3$_USb60min_59kHz samples. Tested in 0.5 M H_2SO_4.*

Subsequently, another comparison emerging from the electrocatalytic tests between optimized samples (synthesis conditions: MnBr's precursor amount equal to 2mL of 5M solution, US source: US bath, sonication time=30min, and US frequency:59kHz), obtained at various calcination temperatures. (**Figure S40**, **Figure S41**, **Figure S42**)

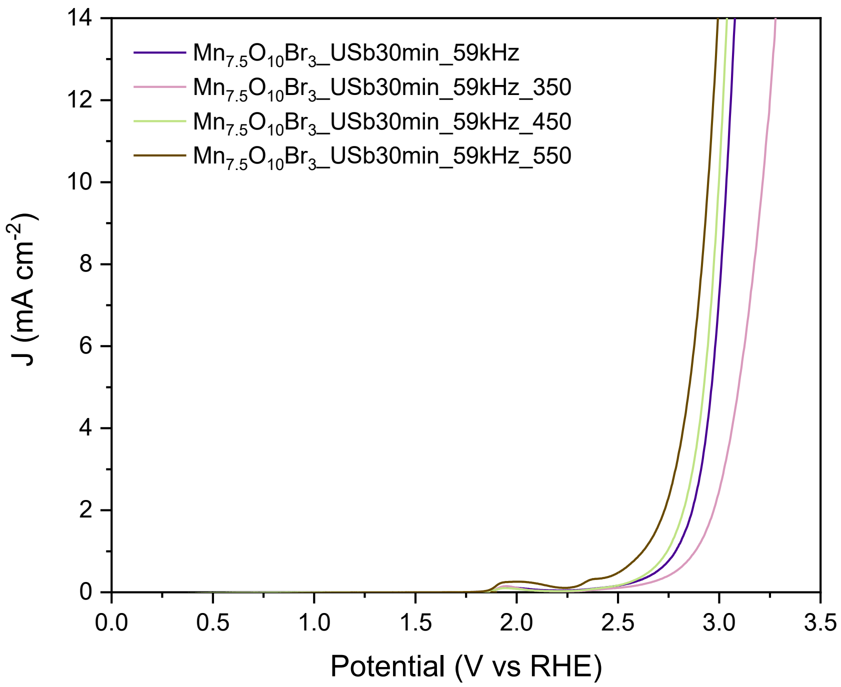

Figure S40. *LSV curves for $Mn_{7.5}O_{10}Br_3$_USb30min_59kHz_250, $Mn_{7.5}O_{10}Br_3$_USb30min_59kHz_350, $Mn_{7.5}O_{10}Br_3$_USb30min_59kHz_450 and $Mn_{7.5}O_{10}Br_3$_USb30min_59kHz_550 samples at 5 mV s^{-1}. Tested in 0.5 M H_2SO_4.*

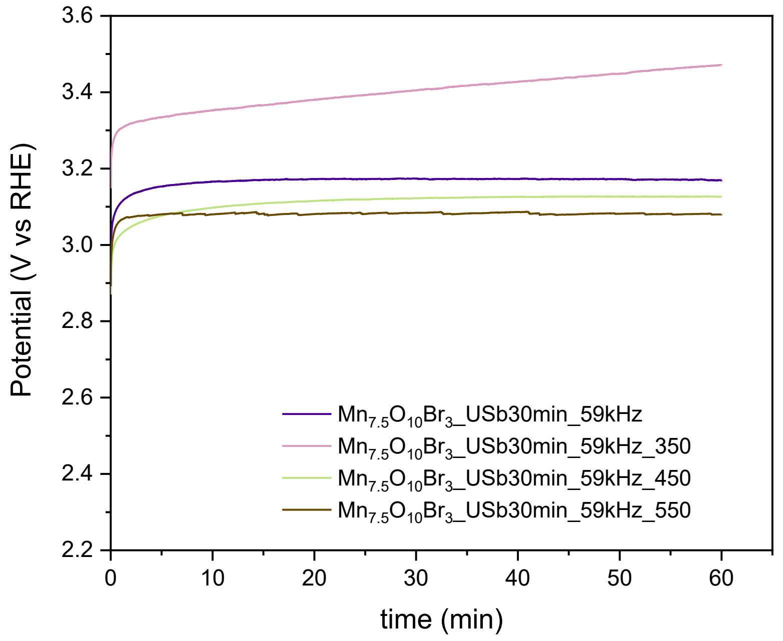

Figure S41. *CP curves for $Mn_{7.5}O_{10}Br_3$_USb30min_59kHz, $Mn_{7.5}O_{10}Br_3$_USb30min_59kHz_350, $Mn_{7.5}O_{10}Br_3$_USb30min_59kHz_450 and $Mn_{7.5}O_{10}Br_3$_USb30min_59kHz_550 samples at 10 mA cm^{-2}. Tested in 0.5 M H_2SO_4.*

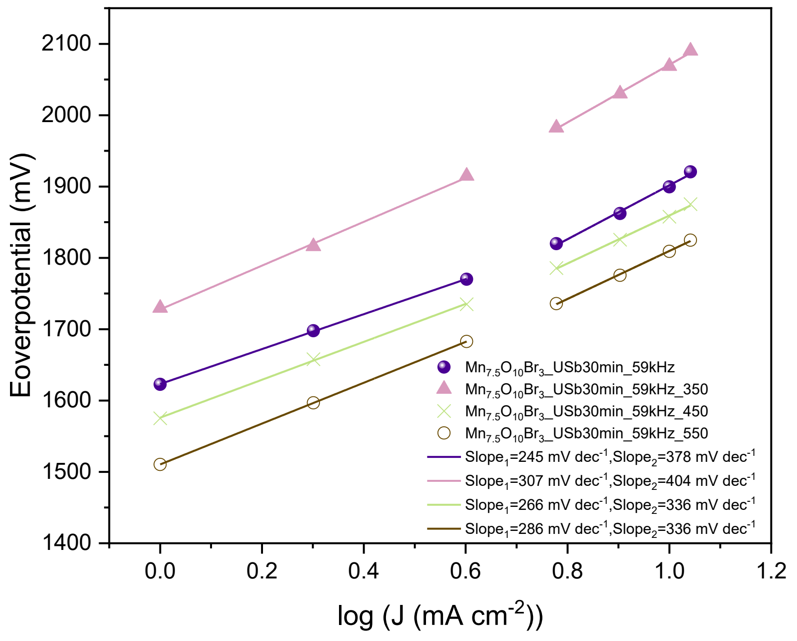

Figure S42. *Tafel Plots for $Mn_{7.5}O_{10}Br_3$_USb30min_59kHz, $Mn_{7.5}O_{10}Br_3$_USb30min_59kHz_350, $Mn_{7.5}O_{10}Br_3$_USb30min_59kHz_450 and $Mn_{7.5}O_{10}Br_3$_USb30min_59kHz_550 samples. Tested in 0.5 M H_2SO_4.*

The performances obtained with the electrode made with $Mn_{7.5}O_{10}Br_3$_USb30min_59kHz on Ti-mesh FTO coated by drop casting (the dark green line) are the best ones among all the electrocatalysts tested in terms of stability and activity, reported in **Figure S43** and **Figure S44**. The bare Ti-mesh FTO coated is reported for comparison, too.

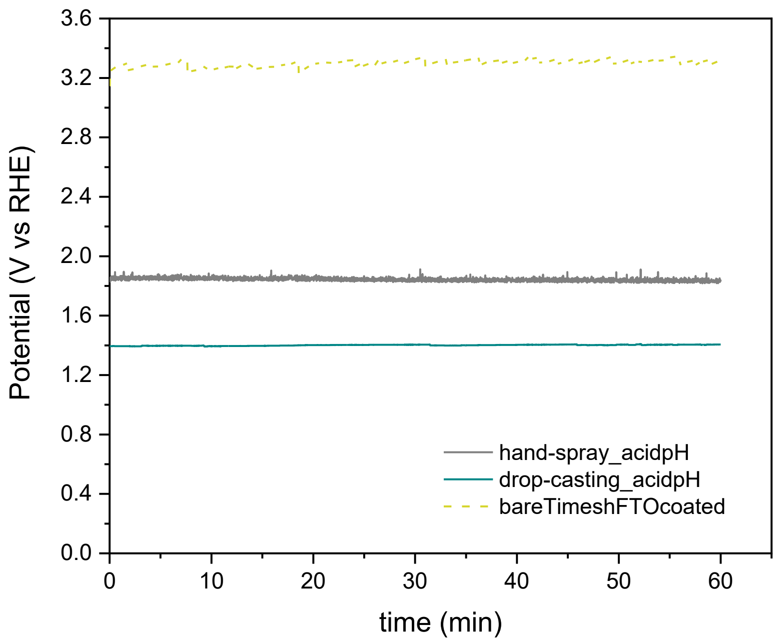

Figure S43. *CP curves for drop-casting_acidpH, hand-spray_acidpH and bareTimeshFTOcoated samples at 10 mA cm^{-2}. Tested in 0.5 M H_2SO_4.*

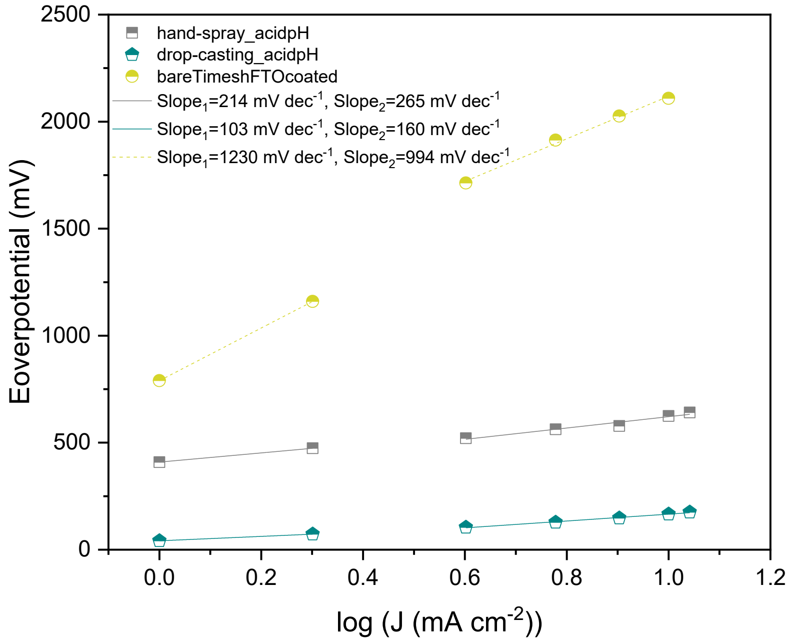

Figure S44. *Tafel Plots for drop-casting_acidpH, hand-spray_acidpH and bareTimeshFTOcoated samples. Tested in 0.5 M H_2SO_4.*

A summary overview, taking the best sample from each group and the ones on Ti-messh FTO coated, is reported below. CP tests (Figure S45) and Tafel slopes graph (Figure S46) show how the effort to improve the catalyst, the electrode and the test led to an important improvement in the electrochemical performances. The values are reported in **Table S11**, too

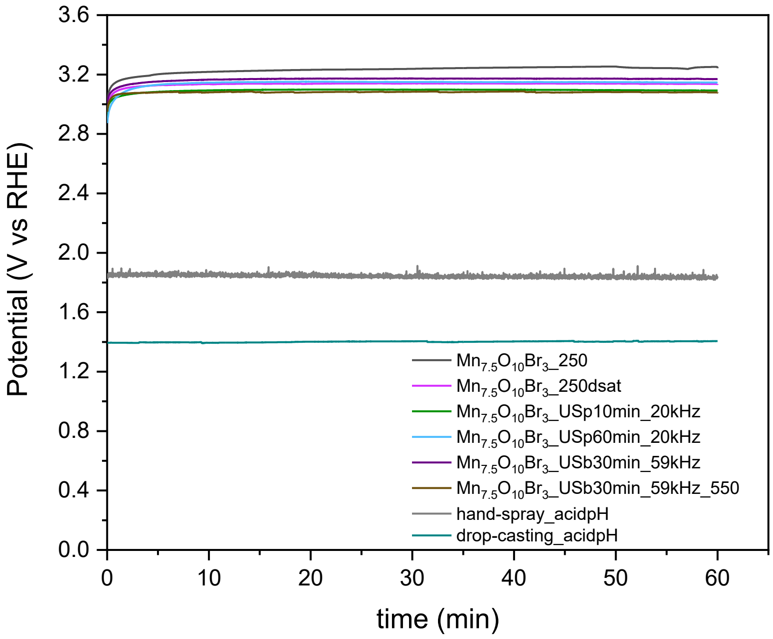

***Figure S45**. CP curves for $Mn_{7.5}O_{10}Br_3$_250, $Mn_{7.5}O_{10}Br_3$_250dsat, $Mn_{7.5}O_{10}Br_3$_USp10min_20kHz, $Mn_{7.5}O_{10}Br_3$_USp60min_20kHz, $Mn_{7.5}O_{10}Br_3$_USb30min_59kHz, $Mn_{7.5}O_{10}Br_3$_USb30min_59kHz_550, hand-spray_acidpH and drop-casting_acidpH samples at 10 mA cm^{-2}. Tested in 0.5 M H_2SO_4.*

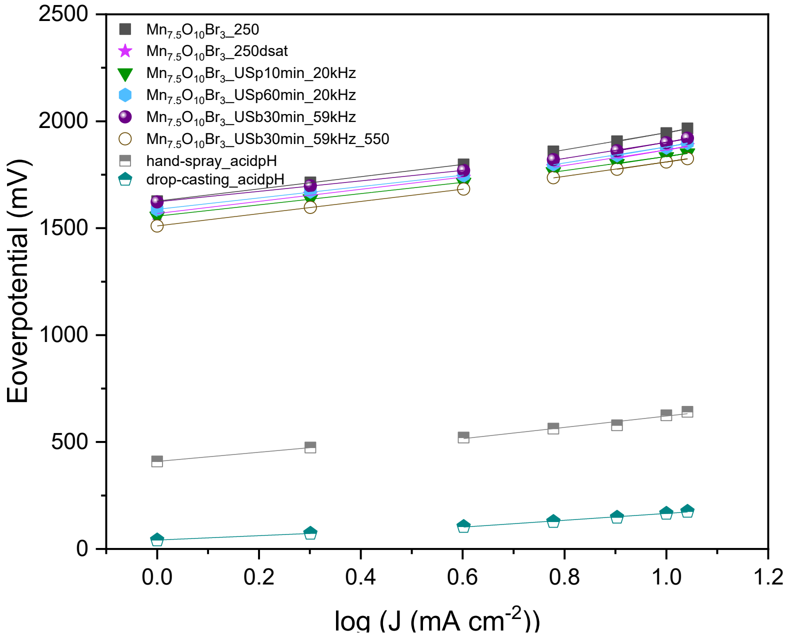

***Figure S46.** Tafel Plots for $Mn_{7.5}O_{10}Br_3$_250, $Mn_{7.5}O_{10}Br_3$_250dsat, $Mn_{7.5}O_{10}Br_3$_USp10min_20kHz, $Mn_{7.5}O_{10}Br_3$_USp60min_20kHz, $Mn_{7.5}O_{10}Br_3$_USb30min_59kHz, $Mn_{7.5}O_{10}Br_3$_USb30min_59kHz_550, hand-spray_acidpH and drop-casting_acidpH samples. Tested in 0.5 M H_2SO_4.*

Table S11. *Overpotential and Tafel slope values for the best sample of each group.*

**Samples**	**Overpotential, η (mV) @ 10 mA cm^{-2}**	**Tafel Slope, b (mV dec^{-1})**	
		$Slope_1$ (low J (mA cm^{-2}))	$Slope_2$ (high J (mA cm^{-2}))
**$Mn_{7.5}O_{10}Br_3$_250**	2003	273	401
**$Mn_{7.5}O_{10}Br_3$_250dsat**	2122	283	365
**$Mn_{7.5}O_{10}Br_3$_USp10min_20kHz**	1903	263	331
**$Mn_{7.5}O_{10}Br_3$_USp60min_20kHz**	2131	266	375
**$Mn_{7.5}O_{10}Br_3$_USb30min_59kHz**	2155	245	378
**$Mn_{7.5}O_{10}Br_3$_USb30min_59kHz_550**	1828	286	336
**Hand-spray_acidpH**	593	215	264
**Drop-casting_acidpH**	153	103	160
